# Genomic Analysis of Antimicrobial Resistance and Resistance Plasmids in *Salmonella* Serovars from Poultry in Nigeria

**DOI:** 10.3390/antibiotics10020099

**Published:** 2021-01-20

**Authors:** Abdurrahman Hassan Jibril, Iruka N. Okeke, Anders Dalsgaard, Vanesa García Menéndez, John Elmerdahl Olsen

**Affiliations:** 1 Department of Veterinary and Animal Sciences, Faculty of Health and Medical Sciences, University of Copenhagen, 1870 Frederiksberg, Denmark; jibrilah50@yahoo.com (A.H.J.); adal@sund.ku.dk (A.D.); vanesag.menendez@usc.es (V.G.M.); 2 Department of Veterinary Public Health and Preventive Medicine, Faculty of Veterinary Medicine, Usmanu Danfodiyo University Sokoto, Sokoto 234840, Nigeria; 3 Department of Pharmaceutical Microbiology, Faculty of Pharmacy, University of Ibadan, Ibadan 234200, Nigeria; iruka.n.okeke@gmail.com; 4 School of Chemical and Biomedical Engineering, Nanyang Technological University, Singapore 637459, Singapore; 5 Laboratorio de Referencia de Escherichia coli (LREC), Departamento de Microbioloxía e Parasitoloxía, Facultade de Veterinaria, Universidade de Santiago de Compostela (USC), 27002 Lugo, Spain

**Keywords:** resistance, plasmids, *Salmonella*, poultry, *Salmonella* genomic islands, whole genome sequence, Nigeria

## Abstract

Antimicrobial resistance is a global public health concern, and resistance genes in *Salmonella*, especially those located on mobile genetic elements, are part of the problem. This study used phenotypic and genomic methods to identify antimicrobial resistance and resistance genes, as well as the plasmids that bear them, in *Salmonella* isolates obtained from poultry in Nigeria. Seventy-four isolates were tested for susceptibility to eleven commonly used antimicrobials. Plasmid reconstruction and identification of resistance and virulence genes were performed with a draft genome using in silico approaches in parallel with plasmid extraction. Phenotypic resistance to ciprofloxacin (50.0%), gentamicin (48.6%), nalidixic acid (79.7%), sulphonamides (71.6%) and tetracycline (59.5%) was the most observed. Antibiotic resistance genes (ARGs) detected in genomes corresponded well with these observations. Commonly observed ARGs included *sul1*, *sul2*, *sul3*, *tet (A)*, *tet (M)*, *qnrS1*, *qnrB19* and a variety of aminoglycoside-modifying genes, in addition to point mutations in the *gyrA* and *parC* genes. Multiple ARGs were predicted to be located on IncN and IncQ1 plasmids of *S.* Schwarzengrund and *S.* Muenster, and most *qnrB19* genes were carried by Col (pHAD28) plasmids. Seventy-two percent (19/24) of *S.* Kentucky strains carried multidrug ARGs located in two distinct variants of *Salmonella* genomic island I. The majority of strains carried full SPI-1 and SPI-2 islands, suggesting full virulence potential.

## 1. Introduction

*Salmonella* is an important pathogen affecting the poultry industry and is of high zoonotic importance [1]. In the USA alone, approximately 1.2 million human cases and 450 deaths occur each year due to infection with this bacterium [2]. In most cases, infected humans show self-limiting diarrhea; however, a life-threatening systemic infection may occur. In such cases, antimicrobial treatment is required [2]. Sadly, in recent years, multidrug-resistant (MDR) *Salmonella* isolates have been encountered with high frequency in many parts of the world, including Europe [3] and sub-Saharan Africa [4], making empiric treatment of salmonellosis difficult.

The development of antimicrobial resistance (AMR) has been attributed to the overuse and misuse of antimicrobials both in veterinary and human medicine, especially in low- and middle-income countries. In Nigeria, overuse of gentamicin, tetracycline, sulphonamides and other antibiotics has been reported due to a lack of a regulatory framework to enforce the law [5]. Food-producing animals, such as poultry, are the main reservoir for *Salmonella* and are important in the development and spread of resistance. AMR might be transmitted to humans via direct contact, or through the food chain and the environment [6]. Resistance genes may also spread from *Salmonella* in food animals to humans via mobile genetic elements (MGEs), such as plasmids, integrons and transposons harboring resistance genes [7]. These mobile elements, in particular plasmids, may spread rapidly from multidrug-resistant (MDR) clones of *Salmonella* within both human and animal hosts [8]. Certain plasmids have been associated with rapid dissemination of resistance in bacteria in clinical settings [9], and some of these plasmids have been termed “epidemic resistance plasmids” due to their high tendency to acquire resistance genes and rapid dissemination among members of *Enterobacteriaceae* [10].

Previous studies have investigated the prevalence of AMR in *Salmonella* in sub-Saharan Africa [11,12,13,14,15]. However, there is scarce information on the role of resistance plasmids in the spread of MDR *Salmonella* in the region, particularly in poultry production, and it is currently unknown whether the so-called epidemic resistance plasmids are harbored in strains of *Salmonella*. This information is essential to control the spread of AMR, as well as to monitor the emergence of new resistant clones. Thus, in this study, we determine the occurrence of AMR in *Salmonella* strains from poultry in Nigeria and determine an association between resistance genes and mobile plasmids using whole genome sequence data. Additionally, we assess the pathogenic potential of the strains by identifying *Salmonella* pathogenicity islands and other virulence factors.

## 2. Results

### 2.1. Phenotypic and Genotypic Resistance

A strain collection consisting of 74 isolates of *Salmonella* belonging to 23 serovars obtained by random sampling in 165 poultry farms in Nigeria was tested (File S1). Phenotypically, all strains showed resistance to one or more of the 11 antimicrobials in the panel tested. Strains showed a high level of resistance to nalidixic acid (79.7%), sulphonamides (71.6%), tetracycline (59.5%), ciprofloxacin (50.0%), kanamycin (50%) and gentamicin (48.6%) (Table 1). Resistances against chloramphenicol (21.6%), trimethoprim (12.2%) and ampicillin (9.4%) were also detected. Interestingly, cephalosporin and meropenem resistance was observed in seven strains and one strain, respectively. Forty-two strains (56.8%) were MDR, and the percentage of strains within each serovar showing MDR can be seen from the last column in Table 1.

All strains of *S.* Schwarzengrund, *S*. Aberdeen, *S*. Essen and *S*. Menston were MDR. In addition, most of the strains of *S.* Kentucky (87.5%), *S.* Muenster (75.0%) and *S.* Isangi (62.5%) were MDR (Table 1). One *S.* Kentucky strain showed resistance to all but one of the 11 antimicrobials tested. One *S.* Larochelle strain showed resistance to meropenem and to seven other antimicrobials. 

In silico predictions, using ResFinder, identified 26 different antimicrobial resistance genes (Table 2). In addition, point mutations in DNA gyrase (*gyrA*; Ser83Phe and Asp87Tyr) and DNA topoisomerase (*parC*; Thr57Ser and Ser80Ile) conferring resistance to nalidixic acid and ciprofloxacin were observed in 70.3% and 35.1% of isolates, respectively. The most commonly predicted resistance genes were combinations of *aac (6′)-Iaa* and *aph (3′)-Ia,b* found in 95.0% of isolates, which confer resistance to kanamycin. Thirty-two isolates carrying the *aac (6′)-Iaa* gene were phenotypically susceptible to kanamycin. Genome analysis revealed that 13 of these isolates had stop codons inserted at a specific position of the promoter region, while 19 possessed several stop codons at different positions of the promoter (Figure S1). Moreover, a total of 45.9%, 44.6% and 37.8% of strains harbored genes which confer resistance to tetracycline (*tet(A)*, *tet(M)*), sulphonamides (*sul1,2,3*) and gentamycin (*aac (3)-Ia, aac(3)-IIa, aac(3)-IVa, aac(6′)-IIa, aac (3)-Id)*, respectively (Table 2). Only few strains (5.4%, 8.1% and 8.1%) were predicted to carry resistance genes to ampicillin (*bla*_TEM_), trimethoprim (*dfrA14, dfrA15, dfrA17*) and chloramphenicol (*catA1, cmlA1, floR*) (Table 2). Surprisingly, no resistance genes or point mutations conferring resistance to colistin were observed. Genes encoding resistance to more than three classes of antimicrobials were observed in *S.* Kentucky, *S.* Isangi, *S.* Schwarzengrund and *S.* Muenster.

A substantial agreement with statistical significance between genotypic predictions and phenotypic resistance to trimethoprim (k = 0.78, *p* = 0.0001), ampicillin (k = 0.7, *p* = 0.0001), tetracycline (k = 0.68, *p* = 0.0001) and gentamicin (k = 0.62, *p* = 0.0001) resistance was observed. Only moderate yet significant agreement was observed for chloramphenicol (k = 0.49, *p* = 0.0001) and ciprofloxacin (k = 0.52, *p* = 0.0001) resistance, and fair agreement was found for nalidixic acid resistance (k = 0.37, *p* = 0.001), while no agreement was observed for kanamycin (k = 0.00, *p* = 1.0) resistance (Table S2A).

When epidemiological cut-off (ECOFF) values were used as cut-offs, there was a reduction in the level of concordance between the phenotype and prediction of resistance genes (Table S2B). In order to understand whether false predictions were associated with strains having zone diameters close to the breakpoint, we analyzed the association between zone diameter and the prediction of genes. The result showed that for all antimicrobials, the group of strains with phenotypic resistance and lack of relevant genes and/or mutations to explain this included both strains with zone diameters close to the breakpoint and strains with very narrow zones, indicating that not all false predictions could be explained by the uncertainty of classification close to the breakpoint. The same was the case for strains which were sensitive to disc diffusion, but where a relevant gene and/or mutation was detected (Figure S2).

### 2.2. Plasmid Replicons and Association with Resistant Genes

Among the 74 isolates, 30 isolates, belonging to seven serovars, contained plasmid replicons predicted by PlasmidFinder. In total, 12 different plasmid replicons were detected, with Col (pHAD28), ColpVC and IncFIIpCRY as the most commonly found replicons. *S.* Schwarzengrund (2/4) and *S.* Muenster (3/4) were predicted to have IncN and IncQ1 plasmids, while *S.* Takoradi (3/6), *S.* Larochelle (4/4) and *S.* Isangi (6/8) were predicted to have IncL, Col (pHAD28)/Col (Ye4449) and Col (pHAD28) replicons, respectively. Strains of *S.* Kentucky showed variation in the plasmid replicons (11/24 positive for replicons) with the presence of IncFIIpCRY, ColpVC and IncM1 replicons (Table 3). Plasmid profiling was run in parallel, confirming the presence of 29 out of 35 in silico predicted plasmids (Table 3). Plasmid profiles of strains of *S.* Muenster, *S.* Schwarzengrund, *S.* Isangi and *S.* Kentucky isolates are shown in Figure S3.

Among the 30 strains with identified plasmid replicons, eight strains contained plasmids predicted to carry multiple antimicrobial resistance genes, while ten strains carried plasmids where the only predicted resistance gene was *qnrB19*. In the remaining 12 strains, no resistance genes were predicted to be located on the putative plasmids.

**Figure 1:**
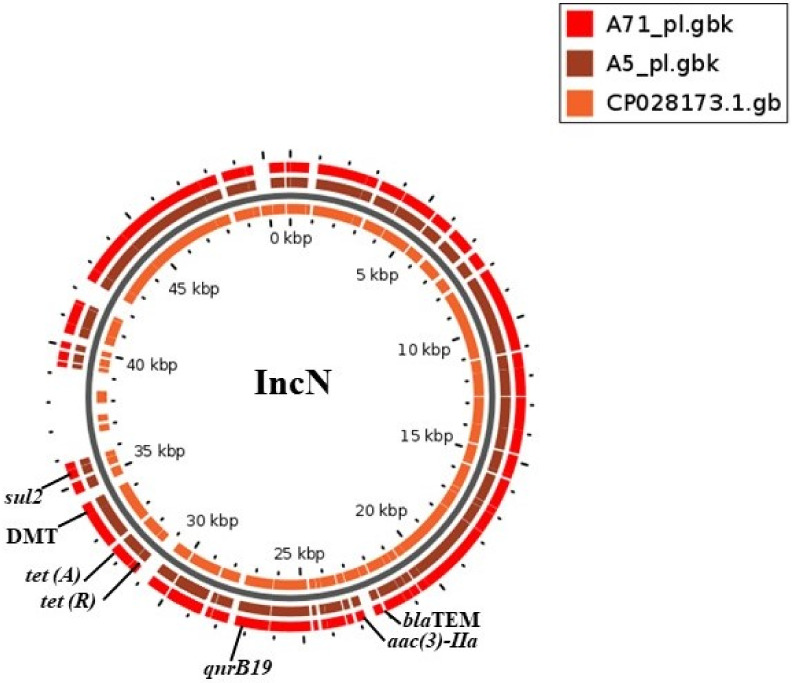
BLAST atlas created using GView of two plasmids from *S*. Schwarzengrund carrying multidrug resistance genes with the reference plasmid CP028173.1. The innermost ring represents the genes of the reference plasmid, while the two outermost rings are the query plasmids from this study. Genomic locations of resistance genes are labeled.

Multidrug resistance IncN plasmids from two strains of *S.* Schwarzengrund were aligned with a reference sequence (CP028173.1) using BLASTN. There was 99.9% identity over 86.0% query cover of these plasmids and the reference sequence (*Salmonella enterica* strain CFSAN064033 plasmid pGMI17-001_1, complete). The resistance genes were flanked by a repeat region, followed by transposase (*Tn3*). The transposase was identified to be homologous to an NCBI reference sequence (pfam01526). Resistance genes harbored by these plasmids were *sul2*, *tet(A)*, *tet(R)*, *aac(3)-II, aph (3”)-I, aph (6)-Ic, qnrB19* and *bla*_TEM_ (Figure 1). These plasmids were also found to have an efflux system (permease of drug/metabolite transporter (DMT)), conjugative transfer proteins (IncN kikA protein, IncQ plasmid conjugative transfer protein, IncW plasmid conjugative protein TrwB), a resolvase/integrase TinR protein, hypothetical proteins, a RepE replication initiator and other plasmid-related proteins (File S2).

**Figure 2:**
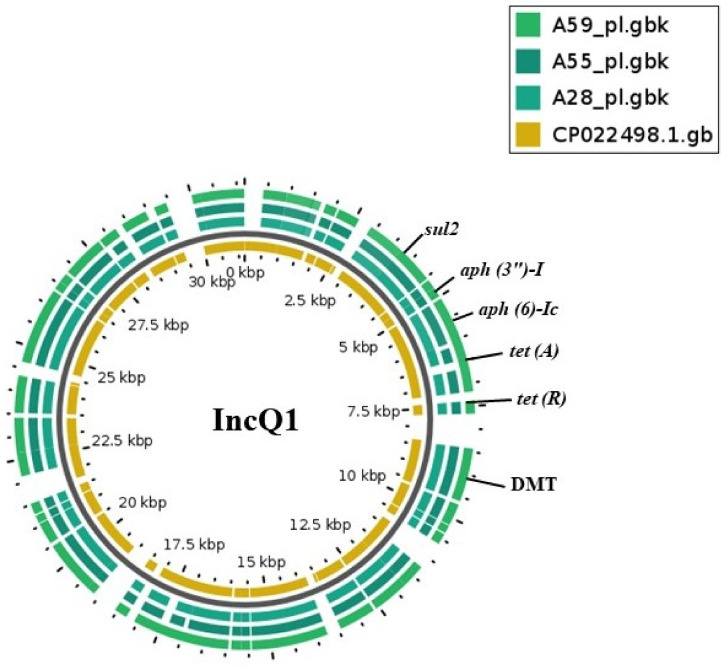
BLAST atlas created using GView of IncQ1 plasmids from *S*. Muenster carrying multidrug resistance genes with the reference plasmid CP022498.1. The innermost ring represents the genes of the reference plasmid, while the three outermost rings are the query plasmids from this study. Genomic locations of resistance genes are labeled. The permease of the drug/metabolite transporter (DMT) superfamily is also shown.

Similarly, all three IncQ1 plasmids from *S.* Muenster were aligned with a reference sequence (CP022498.1). There was 99.9% identity with 94.0% query cover between replicons and reference sequence (*Salmonella enterica* subsp. *enterica* serovar Manhattan strain SA20084699 plasmid unnamed1, complete sequence). The plasmid harbored *sul2, tet(A), tet(R), aph(3″)-I* and *aph (6)-Ic* resistance genes (Figure 2) which were flanked by mobile elements. The mobile element from these plasmids showed 90.0% query cover and 99.9% identity with a mobile element (ISCR2 transposase, MN507533.1) of the *Proteus* genomosp. 6 strain from NCBI BLAST. Additional detected genes were an efflux system (permease of drug/metabolite transporter (DMT)), hypothetical proteins, a relaxase and replication protein (RepA)-encoding genes. The remaining three plasmids with genes responsible for multidrug resistance were one IncM1 plasmid and one IncHI2/IncHI2A plasmid from *S.* Kentucky and one IncHI2/IncHI2A plasmid from *S.* Isangi.

**Figure 3:**
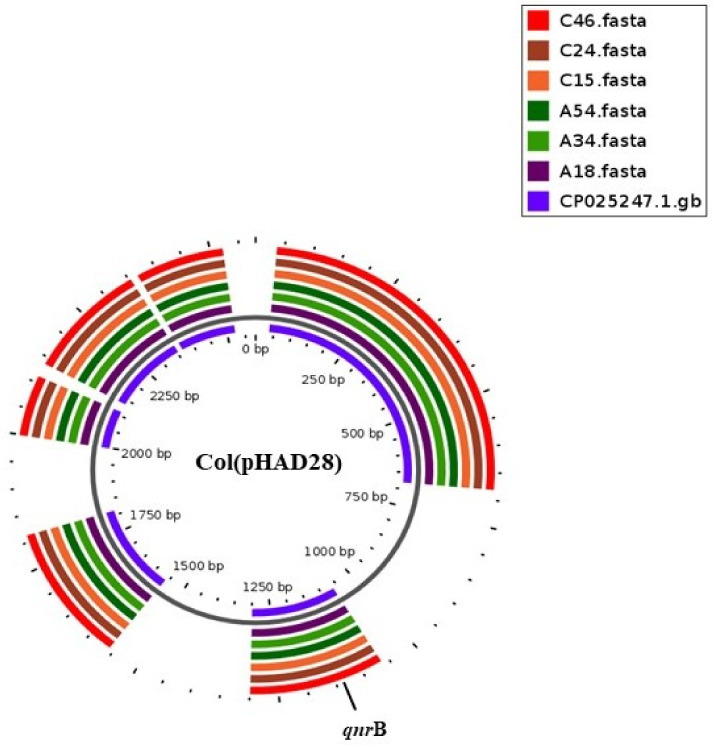
BLAST atlas created using GView of the Col (pHAD28) plasmid from *S*. Isangi carrying a plasmid-mediated quinolone resistance gene (*qnrB19*) with the reference plasmid CP025247.1. The innermost ring represents the genes of the reference plasmid, while the three outermost rings are the query plasmids from this study. Genomic locations of resistance genes are labeled.

Col (pHAD28) plasmids harboring *qnrB* genes from six strains of *S.* Isangi were aligned with reference plasmid CP025247.1 (Figure 3). There was 100% query cover with 100% identity between replicons and reference sequence (*Salmonella enterica* subsp. *enterica* serovar Newport str. CDC 2012K-0938 plasmid pSNE1-2012K-0938, complete sequence). These small-size plasmids harbor genes which reduce susceptibility to quinolones. Other genes located on these plasmids are the *psp* operon transcriptional activator, a putative replicase, hypothetical proteins and an uncharacterized Bsu1883a protein (File S2). The remaining four strains carrying Col (pHAD28) plasmids with *qnrB* genes were four strains of *S.* Larochelle. 

In addition to multidrug-resistant genes, efflux systems for exporting drugs or metabolites were encoded by some plasmids. Seven of these plasmids had a permease of the drug/metabolite transporter (DMT), while one strain each of *S.* Kentucky and *S.* Isangi carried a multidrug efflux pump (Mdtl) and small multidrug-resistant genes (*smr*). Plasmids of the IncHIA/IncHI2A type in one strain of *S.* Kentucky were shown to harbor a resistance gene against a macrolide (*mphA*) (Table S1). 

Eight isolates of *S.* Kentucky carried plasmids bearing no resistance genes. These included four small ColpVC plasmids, which contained replication origin and hypothetical protein genes only. An IncFIIpCRY replicon was present in four strains and was mapped to plasmids which carried *traG, ccdB toxin* and *virB* genes. Furthermore, three IncL plasmids from *S.* Takoradi harbored genes encoding phage proteins, *traI* and *traY* in addition to other encoded genes (File S2). These plasmids, too, were aligned with respective plasmid references from NCBI (Figure S3).

### 2.3. Genomic Islands Bearing Multiple Resistance Genes in S. Kentucky Strains

**Figure 4:**
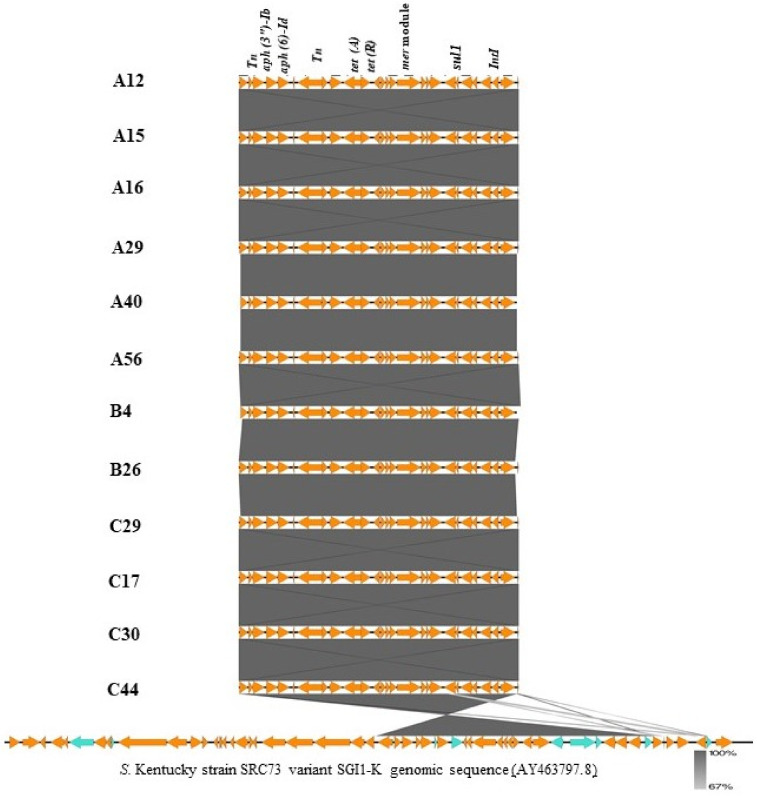
Alignment of 20 kb region of *Salmonella* genomic islands of 12 study strains (cluster I) with the SGI1-K reference sequence, created using EasyFig. Orange arrows represent coding regions of genes; blue arrows represent non-coding regions.

**Figure 5:**
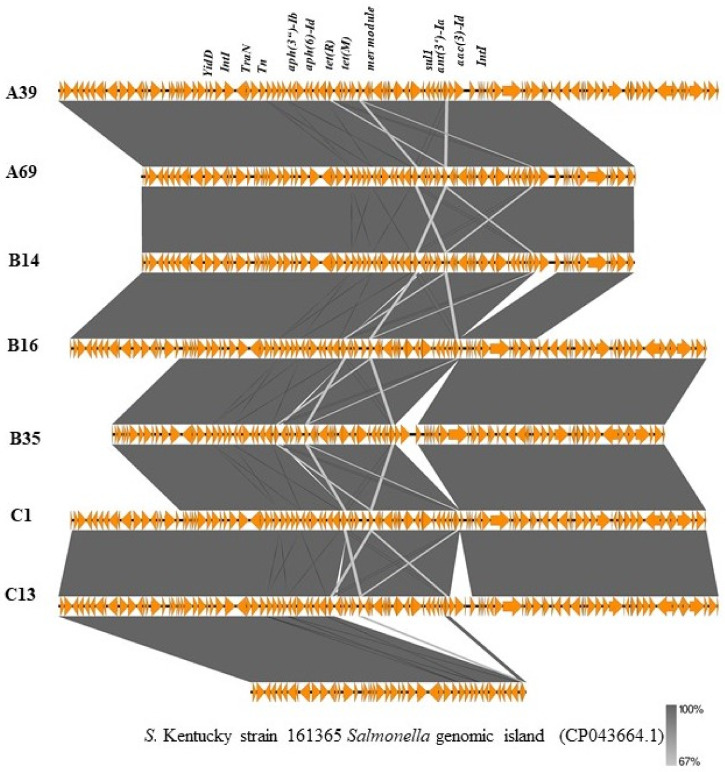
Alignment of 48 kb region of *Salmonella* genomic islands of 7 study strains (cluster II) with different variants of the SGI1-K reference sequence, created using EasyFig. Orange arrows represent coding regions of genes; blue arrows represent non-coding regions.

ResFinder predicted seven resistance genes (*aac (3)-Id, aadA7*, *aph (3″)-Ib*, *aph (6′)-Id*, *tet(A)*, *sul1*) on specific contigs of 19 strains of *S.* Kentucky. From NCBI BLAST, twelve of these strains had these genes located on a region that is homologous to the approximately 20 kb *Salmonella* genomic island 1 variant SGI1-K in *S.* Kentucky strain SRC73 (GenBank accession no. AY463797.8) with 100% coverage and 99.9% identity. Alignment of this cluster I with a reference strain is shown in Figure 4. The remaining seven isolates were also identified to have a 48 kb region that was homologous to a region of a different variant of *S.* Kentucky strain 161,365 *Salmonella* genomic island (GenBank accession no. CP043664.1) with 95% coverage and 100% identity. Alignment of this cluster II with a reference is shown in Figure 5. In addition to resistance genes, the islands possessed an *intI*-encoding integrase, *yid* genes, mercury resistance modules (*mer* genes), *tra* genes involved in conjugative transfer, insertion sequences and other genes involved protein transport and metabolism (File S3).

### 2.4. Virulence Genes

Many of the sequenced strains belong to serovars which are not commonly reported and therefore not studied in detail. In order to assess their potential to cause human disease, genome sequences were searched for known virulence factors. SPIFinder predicted nine different *Salmonella* pathogenicity islands (SPIs) including SPI-1, SPI-2, SPI-3, SPI-4, SPI-5, SPI-8, SPI-13, SPI-14 and centisome 63 pathogenicity island (C63PI). C63PI was present in all strains. Thirty-three strains carried SPI-1, SPI-2 and SPI-3 genomic islands, while seven strains had SPI-1 and SPI-2 only, eleven strains had SPI-1 and SPI-3 only, one strain had SPI-2 and SPI-3 only, four strains had SPI-3 only and eight strains had SPI-1 only. The draft genomes of three *S*. Kentucky strains, two *S.* Isangi strains, two *S*. Telekebir strains, one *S.* Muenster strain, one *S.* Abadina strain and one *S*. Larochelle strain completely lacked the three SPIs. Some variation in gene presence or absence in SPI gene profiles of strains showing the same serovar was observed; however, most strains of a serovar shared identical SPI profiles (Table 4).

Other common virulence factors in the virulence factor database (VFDB) shared by most serovars included fimbria and non-fimbrial adherence determinants, a macrophage-inducible gene (*mig-14*) and the *phoP* and *phoQ* regulatory genes. Strains were observed to have different profiles of SPI-1 and SPI-2 effectors genes (File S4). It is worth noting that 19 of the strains carried genes that encode for cytholethal distending toxins (*cdtB, pltA, pltB*). This included all four strains of *S.* Schwarzengrund, three of *S.* Muenster, five of *S.* Takoradi, two of *S.* Chester and one each of *S*. Ituri, *S.* Give, *S.* Poona and *S.* Telekebir. The complete virulence gene profile of each strain is shown in File S4.

## 3. Discussion

Antimicrobial resistance (AMR) in zoonotic and foodborne pathogens is considered a serious threat to public health [16]. The rapid emergence and pandemic spread of AMR is particularly worrisome in non-typhoidal *Salmonella* (NTS), one of the most common causes of foodborne disease and an important cause of mortality worldwide [16]. *Salmonella* is endowed with virulence factors that allow systemic propagation leading to severe infections, particularly in immunocompromised patients, and it has the ability to rapidly acquire antimicrobial resistance genes from related and unrelated bacteria, especially intestinal commensals [17]. In the present study, we used phenotypic and genotypic methods to characterize antimicrobial resistance in *Salmonella* from poultry in Nigeria, the largest poultry producer in sub-Saharan Africa [18]. We used genomic data to infer the virulence potential of the strains to cause infections in humans. A pool of resistance genes introduced into the human intestine with *Salmonella* may also be problematic because such genes may spread to other bacteria; to assess the likelihood of this, we analyzed whole genome sequencing data to determine whether resistance genes were carried on transferrable plasmids.

All strains were resistant to at least one antimicrobial, and more than 50% displayed MDR; in particular, strains of serovars *S.* Kentucky, *S.* Isangi, *S.* Schwarzengrund, *S.* Muenster and *S.* Telekebir showed MDR. All strains were predicted to have at least one ARG. A different degree of concordance was observed between predictions of ARGs and resistance to a specific class of antimicrobial. The level of concordance reduced when ECOFF was used as a cut-off point compared to when clinical breakpoints were used. This could be attributed to the fact that ECOFF values for disk diffusion methods are often set higher than the cut-off values for clinical breakpoints, and there is the likelihood of classifying a resistant (non-wild-type) strain as sensitive (wild-type). Misclassification of resistance could also be attributed to the fact that clinical breakpoints include intermediate classification ranges that reflect the concentration range for which isolates may contain resistance determinants but do not present zone diameters above the resistance threshold [19]. Zone diameters in strains with phenotypic resistance and a lack of a relevant ARG varied considerably, and some strains had narrow zone diameters. Such strains are likely to carry a resistance mechanism not included in the databases used in the current study. Other strains in this group had zone diameters close to the breakpoint on the narrow side, and it is likely that such strains are misclassified by the disc diffusion method. For example, we detected resistance to cefotaxime in three strains without concurrent resistance to ampicillin. We observed that those strains had zone diameters close to the breakpoints, and probably the classification as resistant to cefotaxime was false. In agreement with this, no ESBL genes were present in these strains. This corresponds well with the fact that β-lactams are not reported to be used in poultry production in Nigeria [5,20]. Another factor which could contribute to false predictions was the use of assembled genomes for resistance prediction. It has previously been shown that the use of raw reads performs better than the use of assembled genomes for this purpose [21]. Furthermore, a strain carrying a resistance gene may fail to express the gene due to a lack of mechanisms necessary for expression of the gene.

Resistance to ampicillin was rare, and *bla*_TEM_ genes were only detected in three strains of *S*. Schwarzengrund and one strain of *S*. Kentucky. This is considerably less frequent than reports from other sub-Saharan countries [11,22]. Production of plasmid-mediated β-lactamases by bacteria is one of the most widespread mechanisms of resistance, and it is reported to be of critical concern to human health. Interestingly, seven strains showed phenotypical resistance to third-generation cephalosporins (cefotaxime); however, these isolates were negative for genes conferring resistance to cephalosporin and ESBL genes were not detected. Further studies are needed to determine which genes encode resistance in these strains. 

Forty-eight percent and 50% of strains were phenotypically resistant to gentamicin and kanamycin, respectively. Prediction of resistance genes to gentamicin (*aac (3)-Ia*, *aac(3)-IIa*, *aac(3)-Iva*, *aac(6′)-IIa* and *aac (3)-Id*) was concordant with phenotypic resistance, while there was slight to no agreement with occurrence of *aac (6′)-Iaa, aph(3′)-Ia,b* and resistance to kanamycin, tobramycin or amikacin [23]. *aac (6)-Iaa* are chromosomally located genes in *Salmonella*, and they may be rendered silent by mutation in the promoter [24]. In accordance with this, we observed stop codons in 43.2% of isolates at different positions of the promoter. However, we detected two isolates that had no mutation in the promoter region of this gene, and which also did not show phenotypic resistance to kanamycin. It is possible that these isolates lack other components necessary to transfer an acetyl group that is required for the resistance mechanism of kanamycin, and further studies are needed to understand the lack of kanamycin resistance in these strains. Other aminoglycoside genes (*aph* *(3″)-Ib, aph (6′)-Id, aadA1*, *aadA5*) that confer resistance to streptomycin and spectinomycin [22] were also predicted. Particularly strains of *S.* Kentucky, *S.* Isangi and *S.* Schwarzengrund displayed resistance to aminoglycosides both phenotypically and genotypically. This finding corroborates several reports demonstrating the presence of multiple aminoglycoside resistance genes in *Salmonella* serovars [24,25,26].

Fifty percent and 79.7% of isolates showed phenotypical resistance to ciprofloxacin and nalidixic acid, respectively. Predictions by ResFinder demonstrated that most of these strains carried either *qnrB19* or *qnrS1* genes with or without the presence of point mutations in DNA gyrase and topoisomerase. The *qnrB19* and *qnrS1* genes encode transferable (fluoro) quinolone resistance mechanisms that are responsible for reduced susceptibility to quinolones [27] and which may enhance clinical resistance to quinolones [28]. Clinical resistance is caused by a point mutation in the quinolone resistance-determining region (QRDR) of DNA gyrase A (*gyrA*) and topoisomerase C (*parC*) genes in most members of Enterobacteriaceae [27]. An increase in the number of mutations leads to a stepwise increase in the level of resistance to ciprofloxacin, while one point mutation in *gyrA* results in resistance to nalidixic acid only [27]. In our study, predominantly *S.* Kentucky, *S*. Isangi, *S*. Schwarzengrund and *S.* Muenster strains showed phenotypic and genotypic resistance to quinolones. These findings corroborate an increasing number of reports revealing that *S.* Kentucky, *S*. Schwarzengrund, *S*. Isangi and *S*. Muenster from livestock carry *qnrB19* and multiple mutations in *parC* and *gyrA* genes [29,30,31]. 

Fifty-three (71.6%) strains were phenotypically resistant to sulphonamides, while only 12% of strains showed resistance to trimethoprim. Likewise, 44.6% and 8.1% of strains were predicted to have sulphonamides and trimethoprim resistance genes, respectively. As previously reported, resistance to folic acid inhibitors is one of the most frequent mechanisms of bacterial resistance to sulphonamides and trimethoprim due to the acquisition of dihydropteroate synthase and a reductase enzyme encoded by the genes *sul1*, *sul2* and *sul3* and *dfrA14, dfrA15* and *dfrA17*, respectively [32]. Predominantly, *S*. Isangi, *S.* Kentucky, *S*. Schwarzengrund and *S.* Muenster strains showed phenotypic and genotypic resistance to sulphonamides, while only *S.* Kentucky and *S.* Isangi carried resistance genes against trimethoprim. Phenotypic resistance to chloramphenicol and tetracycline was mostly observed in strains of *S*. Isangi, *S.* Kentucky and *S*. Schwarzengrund, which also carried genes encoding resistance to these two classes of antimicrobials. Interestingly, genes that confer resistance to macrolides (*mef(B*), *mph(A)*, *erm(B)*) were located in one strain each of *S.* Kentucky, *S.* Chester and *S.* Larochelle, while a resistance gene to fosfomycin (*fosA7*) was found in *S.* Menston. This finding is indeed worrisome because these are broad-spectrum antimicrobials that are mostly used as a drug of last resort in treating human infections. Others have also showed increased resistance to macrolides [33] and fosfomycin resistance [34] in NTS. A strain of *S.* Larochelle was found to be phenotypically resistant to meropenem; however, genomic analysis did not reveal any genes known to encode carbapenem resistance. The lack of genotypic detection could be due the presence of other mechanisms of carbapenem resistance such as overproduction of AmpC β-lactamase, mutation in porin or drug efflux [35]. Interestingly, in this strain, we observed point mutations in the *ompR* porin gene, which could be responsible for the phenotype observed [36]. Other studies have reported phenotypic expression of meropenem resistance and a lack of genotypic detection of *bla*_KPC_ [37,38]. 

Despite reports of high usage of colistin in poultry in Nigeria [20], no genes nor point mutations conferring resistance to this drug were predicted in the strain. Plasmid-encoded colistin resistance may be carried on small plasmids [39], which can be missed in short-read sequencing. However, it is unlikely, that we missed genes due to this reason, since small plasmids encoding other resistances were detected.

Several approaches were used to reconstruct plasmids from whole genome sequence data. This work was conducted in parallel with plasmid extraction. Even though there are limitations in using short-read sequences in plasmid identification, it has been recommended for plasmid analysis [40]. Studies have validated the use of whole genome sequence data for plasmid identification and resistance gene prediction in plasmids [41,42], with a high level of association between in silico predictions and plasmid extraction [43]. To overcome the limitations of the short-read sequence approach, we performed a three-step approach that allowed for plasmid reconstruction. Firstly, we detected plasmid replicons using PlasmidFinder from the CGE server. Then, we used PlasmidSPAdes for assembling those raw sequences containing plasmid replicons. Finally, plasmid components obtained from PlasmidSPAdes were blasted in NCBI, from which we selected hits with at least 99% identity and 99% query cover as a reference for mapping. This approach can overcome the limitation of short reads and has been used recently by others [43]. Extracted plasmids from isolates and electrophoretic band sizes corresponded to the size estimated from PlasmidSPAdes outputs. This further confirmed a strong association (82.9%) between predictions and profiling of plasmids. 

Plasmid replicons were detected in 40% of strains representing seven of the 23 serovars detected. The high proportion of strains where plasmid replicons were not detected was surprising compared to other studies [7,23]. There was homogeneity between putative plasmid types and serovars, in the sense that particular Inc groups were associated with particular serovars. This is in agreement with previous reports that demonstrated the presence of specific incompatibility plasmids in certain serovars [23,43]. Most of the resistance genes located on plasmids were encoded on plasmids of type IncN, IncQ1 and Col (pHAD28) and replicons of these types were harbored by strains of *S.* Schwarzengrund, *S.* Muenster and *S.* Isangi. Especially the IncQ1 of *S.* Shwarzengrund and IncN of *S.* Muenster were found to have multiple resistance genes (*sul2, tet(A), tet(R), aph(3”)-I* and *aph (6)-Ic*) flanked by a repeat region with the *Tn3* transposase. These resistance genes were likely acquired by transposition into this plasmid. In addition to transposase, the plasmid also harbored conjugative transfer genes, demonstrating mobility potential. However, a conjugation assay would be needed to provide insight into the transferability of the resistance genes. Reports of multiple antimicrobial resistance genes, including *bla*_TEM_*,* carried on similar but not identical plasmids in *S.* Enteritidis have previously been reported [7,23,43,44]. IncQ1 plasmids have been associated with MDR in *S.* Typhimurium [45]. Col (pHAD28) plasmids were detected in *S.* Isangi. These are small plasmids, and our study confirms that they harbor *qnrB19* genes, which have been associated with plasmid-mediated reduced susceptibility to quinolones in *Salmonella* Hadar [46]. Plasmid-mediated quinolone resistance has contributed to the rapid increase in and spread of reduced susceptibility to fluoroquinolones among enteric pathogens [26]. Additionally, an appreciable number of them (22.9%) carried genes encoding innate antimicrobial efflux transporters. These are small permeases of the drug/metabolite transporter superfamily (*dmt*), a multidrug efflux pump (*mdtl*) and a small multidrug resistance (*smr*) efflux transporter and are considered the first line of bacterial defense against antimicrobials, by decreasing the intracellular level of the drug [47]. The fact that the strains carrying these plasmids were present in different serovars and were obtained from different farms may indicate the spread of plasmids and possibly that humans and animals may act as vectors for the spread between farms.

Notably, most (91.7%) *S.* Kentucky strains were phenotypically and genotypically resistant to several antimicrobials. Insightfully, 90% of plasmids associated with these strains were predicted not to have ARGs. The most striking finding was that 50% of strains had multiple resistance genes located on regions homologous to the 20 kb region of SGI1-K variants [48], while 29.2% of the others had a region homologous to the 48 kb region of a recently described variant of SGI1 [49]. The islands-contained resistance genes were *aac (3)-Id, aadA7*, *aph (3″)-Ib*, *aph (6′)-Id*, *tet(A)* and *sul1* that confer resistance to gentamicin, spectinomycin, streptomycin, tetracycline and sulphonamide, respectively. The set of genes present in these strains is, however, different from that reported from a previous study [49], which may be attributed to the plasticity that allows for loss or gain of segments by homologous recombination and which is responsible for the development of several variants of SGI1 in *Salmonella* [50]. This finding demonstrates that two major sub-clusters of multidrug-resistant *S.* Kentucky strains were circulating in the study area.

With the exception of *S.* Kentucky, the serovars found in poultry in Nigeria have not been well characterized, and little is known about their potential to cause human disease. SPIs are involved in *Salmonella* adherence, invasion, replication and survival within the host cells [51]. We found variation in SPI profiles between the serovars which indicates differences in virulence potential [51]. SPIs 1, 2 and 3 were observed in most strains; however, *S*. Abadina, *S.* Birmingham and *S.* Poona lacked all these major pathogenicity islands and may therefore be less pathogenic. SPI-1 encodes a Type III secretion system (TSSS) considered essential for *Salmonella* invasion and for growth in the intestine [51,52]. The secretion system translocates effector proteins into host cells to modulate their function [53], and these effector proteins are responsible for induction of inflammation within the host gut in the early stage of infection [16]. Whether *Salmonella* serovars depend on induction of inflammation to grow in the gut of chickens remains to be investigated, and it may be that serovars such as *S*. Abadina, *S.* Birmingham and *S.* Poona have adapted to grow in the gut environment in the absence of inflammation. Phenotypes associated with the presence of T3SS encoded by genes of *Salmonella* pathogenicity island 2 (SPI-2) are considered essential for the development of systemic disease including proliferation of *Salmonella* in host macrophages [54]. SPI-2 was present in 54.1% of strains which may be able to cause systemic disease, particularly in people with a weak immune response. The *phoP/phoQ* regulatory genes which are present in most Gram-negative bacteria were also located in all of our strains. These genes control SPI-2 and, consequently, the expression of virulence genes located in this island [55]. Most strains also harbored SPI-3, which contains genes required for intramacrophage survival and growth in low Mg^2+^ concentrations and for long-term attachment to host cells [51,56]. In addition, all strains were shown to carry the centisome 63 pathogenicity island (C63PI). Recent reports, have demonstrated the presence of this pathogenicity island in most *Salmonella* strains [28,57]. C63PI contains genes required for iron acquisition by *Salmonella* and has been reported to have a nucleotide composition that is significantly different from that of SPI-1 [58].

Reports have shown that *Salmonella* serovars carry different types of chaperone-usher fimbriae, and such fimbriae have been proposed as an additional mediator for allowing some serovars to colonize and persist in different hosts/environments [56,59]. These virulence factors are used for initial attachment to the host enterocytes. Initial attachment to host intestinal mucosa after oral infection is one of the most important stages during *Salmonella* pathogenesis [51]. All strains were shown to carry at least one of the fimbriae adherence determinant genes (*csg, bcf, fim, lpf, pef, saf, sti-k* and *tcf*). There were, however, variations in the specific sub-types of these genes between serovars, and all strains lacked the *sef* and *stg* fimbrial genes. Interestingly, some strains (*S*. Muenster, *S.* Chester and *S.* Aberdeen) were also observed to possess the type six secretion system (T6SS), which might have been acquired through horizontal gene transfer. There seems to be little information available about the association of these serotypes with T6SS. T6SS systems in *Salmonella* are encoded from the horizontally acquired islands, SPI-6 or SPI-19 [60,61]. Reports have demonstrated that T6SS plays a role in *Salmonella* pathogenicity; SPI-19 plays a role in killing competing bacteria in the intestine [61,62], and it would be interesting to investigate the role of T6SS in *S*. Muenster, *S.* Chester and *S.* Aberdeen. 

Interestingly, three *S.* Isangi, *S.* Takoradi and *S.* Larochelle strains were identified to have virulence genes not possessed by the other strains in our study; virulence genes that have not been reported in these serotypes previously. This included genes that encode for toxin (*hlyA, hylA/clyA, cysC1*) production, serum resistance (*rck*), quorum sensing (*luxS*) and biofilm formation (*adeG*) [63,64,65]. A recent study showed that a number of NTS serovars carried genes encoding cytolethal distending toxins (*cdtB*, *pltA*, *pltB*) [66], and the current study adds *S.* Schwarzengrund, *S.* Chester, *S.* Muenster, *S.* Ituri, *S.* Give, *S.* Takoradi and *S.* Telekebir to this list. The *rck* gene is known to confer serum resistance to complement killing by binding human complement factor H and may also contribute to autoaggregation [67]. The prediction of virulence genes using in silico tools should be viewed with care, since no phenotypic indication of virulence was investigated.

## 4. Materials and Methods

### 4.1. Bacterial Strains and Draft Genomes

Seventy-four draft genomes of *Salmonella* isolates belonging to 23 serovars obtained from a previous study were analyzed [68]. Isolates were recovered from 558 shoe-socks and dust samples from 165 randomly selected commercial broilers and layers farms in North West Nigeria between June 2018 and May 2019 [68]. Strains were sequenced on an Ilumina Miseq using paired-end chemistry (2 × 250-bp) (Illumina, San Diego, California, USA). Raw reads were assembled using CGE SPAdes (https://cge.cbs.dtu.dk/services/SPAdes/), and assembled contigs were used to assign serovars to strains using in silico typing resources (SeqSero2 and SISTR). Sequence quality based on total length, N50, contig size and Guanine-Cytosine (GC)content was used to select for strains to be included in the study. Draft whole genome sequences of strains are available from the European Nucleotide Archive under study accession number PRJEB37477 with individual strain accession numbers. Detailed information of strains is shown in File S1.

### 4.2. Antimicrobial Screening and In Silico Prediction of Resistance Genes

Strains were phenotypically tested for susceptibility to 11 commonly used antimicrobials representing eight classes of antimicrobials by the Kirby–Bauer disk diffusion test using Clinical and Laboratory Standards Institute (CLSI) protocols [69]. Antimicrobials tested were ampicillin (10 µg), cefotaxime (30 µg), chloramphenicol (30 µg), ciprofloxacin (5 µg), gentamicin (10s µg), kanamycin (30 µg), meropenem (10 µg), nalidixic acid (30 µg), sulphonamides (300 µg), tetracycline (30 µg) and trimethoprim (5 µg) (Oxoid, UK). The reference strain *Salmonella enterica* subsp. *enterica* serovar Typhimurium ATCC 14,028 was used for quality control purposes. Inhibition zone data were entered into WHONET version 5.6 configured with the tested antimicrobials. Isolates were categorized as sensitive, intermediate or resistant using CLSI clinical breakpoints and CLSI guidelines for disc diffusion. For comparison, available ECOFF values of antibiotics in EUCAST (www.EUCAST.org) were used to classify isolates as wild-type (WT) or non-wild-type (NWT). In this study, WTs are considered as sensitive, while NWTs are referred to as resistant. Strains that were resistant to at least one drug in at least three different antimicrobial classes were defined as multidrug-resistant (MDR) according to [70].

A draft assembled genome was used for prediction using ResFinder v.3.2 with default settings (90% selected percentage identity threshold and 60% selected minimum coverage length), available at the Centre for Genomic Epidemiology (https://cge.cbs.dtu.dk/services/ResFinder/). This online platform uses a curated database of resistance genes to identify acquired antimicrobial resistance genes and chromosomal mutations in total or partial sequences. MEGA X v.10.2.2 was used for the alignment of the *aac (6)-Iaa* gene to identify changes in the sequences that could explain the lack of kanamycin phenotypic expression in 32 isolates carrying the gene [71].

### 4.3. Plasmid Extraction, In Silico Prediction and Reconstruction

A three-step procedure was used to reconstruct plasmids (Figure S5). In the first step, CGE SPAdes v.3.9 (https://cge.cbs.dtu.dk/services/SPAdes/) was used to assemble raw reads into different contigs containing chromosomal and extra-chromosomal sequences. Subsequently, draft genomes were submitted to PlasmidFinder v.2.0 available at the Centre for Genomic Epidemiology (https://cge.cbs.dtu.dk/services/PlasmidFinder/), using default settings (95% selected minimum threshold for percentage identity and 60% selected minimum coverage length). The pipeline uses sequence similarity of replicons to predict putative plasmids. In the second step, raw reads of strains that harbored plasmid replicons were used to extract plasmid contigs using PlasmidSPAdes v.3.13.0. The algorithm in PlasmidSPAdes is able to predict contigs that belong to a plasmid and assign those contigs into components; putative plasmids from the same incompatibility group are assigned into same component [72]. The quality of plasmid contigs was evaluated using QUAST v.5.0.2 [73]. Plasmid contigs were submitted to RAST v.2.0 (https://rast.nmpdr.org/) for annotation and detection of resistant genes and were again submitted to PlasmidFinder v.2.0 to verify previous predictions. In the third step, putative plasmids were used to search for the most similar sequences in the NCBI database using nucleotide BLAST (www.ncbi.nlm.nih.gov). Homologous reference sequences (.gbk) with query strains were downloaded from GenBank. Lastly, to confirm in silico plasmid prediction, plasmid extraction was performed using the [74] protocol. Electrophoresis was carried out on 0.6% agarose gels for three to four hours at 100 V and plasmids obtained from *E. coli* V517 [75] and *E. coli* 39R861 [76] were used as size standards.

### 4.4. Mapping of Resistance Genes on Plasmids and Genomic Islands

To align and display the location of resistant genes on the plasmid, BLAST ring analysis of annotated plasmids with the NCBI reference plasmids was performed in GView v.1.7 server (https://server.gview.ca/guide). Non-resistance plasmids were aligned using progressive MAUVE v.2.4.0 [77]. Specific contigs carrying multiple ARGs predicted from ResFinder v.3.2 in *S*. Kentucky strains, which were not located on plasmids, were BLASTed to NCBI to detect the genomic location of resistance genes. These contigs were aligned with NCBI references using EasyFig v.2.2.3 [78]. 

### 4.5. Identification of Salmonella Virulence

The *Salmonella* pathogenicity island finder (SPIfinder v.1.0; Centre for Genomic Epidemiology (https://cge.cbs.dtu.dk/services/SPIFinder/)) was used to identify SPIs using default settings (95% identity threshold and 60% selected minimum coverage length). To further identify virulence factors, draft genomes were submitted to the virulence factor database (VFDB; http://www.mgc.ac.cn/VFs/). VFanalyzer v.2019 uses orthologous groups to predict virulence genes by conducting iterative BLAST sequence similarity searches. 

### 4.6. Data and Statistical Analysis

Phenotypic and genotypic resistance data were stored in Microsoft Excel 2016 (Microsoft Corporation, Redmond, WA, USA) and later exported to SPSS version 26 (IBM, USA) for inferential statistics. Cohen’s kappa statistic was used to assess the level of concordance between phenotypic resistance and in silico gene predictions. Cohen’s kappa is a statistic measure that is used to evaluate the level of agreement between two test protocols, assigning a kappa value between zero and one. Values ≤ 0 indicate no agreement; 0.01–0.20 none to slight; 0.21–0.40 fair; 0.41–0.60 moderate; 0.61–0.80 substantial; and values in the range of 0.81–1.00 indicate values with an almost perfect agreement [79].

## 5. Conclusions

This study demonstrated that MDR was common in strains of *S.* Kentucky, *S.* Schwarzengrud, *S.* Muenster and *S.* Isangi circulating in commercial poultry farms in Nigeria. Resistance genes were found to be harbored on plasmids of several incompatibility groups, which have previously not been associated with these particular serovars. Two major sub-populations of *S.* Kentucky strains with two variants of *Salmonella* genomic islands, each carrying multiple resistance genes, were identified. The fact that resistance genes were found on mobile elements increases the public health risks, as resistances may not only be transferred from animals to human through infection with *Salmonella*, but also through horizontal spread of plasmids involving intermediate bacterial hosts. Most of the strains were found to possess virulence genes that are essential for human infections, indicating zoonotic potential.

## Figures and Tables

**Table 1:** Phenotypic antimicrobial resistance in *Salmonella* serovars from commercial poultry farms in Nigeria.

Serovar	N	Number of Isolates Resistant to Antimicrobials *											MDR **	
		AMP	GEN	KAN	CTX	CIP	SUL	TET	CHL	TMP	NAL	MEM	N	(%)
Abadina	2	0	0	0	0	0	0	0	0	0	2	0	0	0
Aberdeen	1	0	0	0	1	1	1	1	0	0	1	0	1	100
Alachua	1	0	0	0	0	0	0	0	0	0	1	0	0	0
Birmingham	1	0	0	0	0	0	0	0	0	0	0	0	0	0
Bradford	1	0	0	0	0	0	1	0	0	0	1	0	0	0
Chester	2	0	1	2	0	1	2	1	0	0	1	0	1	50
Chomedey	1	0	0	0	0	0	1	1	0	0	0	0	0	0
Colindale	1	0	0	0	0	0	1	0	1	0	0	0	0	0
Corvalis	2	0	1	1	0	1	1	1	0	0	1	0	1	50
Essen	1	0	0	1	0	0	1	1	1	0	1	0	1	100
Give	1	0	0	0	0	0	0	0	0	0	1	0	0	0
Isangi	8	0	5	2	0	5	4	5	5	5	8	0	5	62.5
Ituri	2	0	0	0	0	0	2	1	0	0	2	0	0	0
Kentucky	24	3	21	21	4	23	21	22	3	1	23	0	21	87.5
Larochelle	4	0	1	1	0	2	1	1	1	1	4	1	2	50
Menston	1	0	0	0	0	0	0	1	1	1	1	0	1	100
Muenster	4	0	0	1	1	1	4	3	1	0	4	0	3	75
Poona	1	0	1	0	0	0	1	0	0	0	0	0	0	0
Schwarzengrund	4	4	4	2	1	1	4	4	2	0	4	0	4	100
Takoradi	6	0	1	4	0	1	4	1	0	0	1	0	1	16.7
Telelkebir	3	0	1	2	0	1	2	1	1	1	2	0	1	33.3
Virchow	1	0	0	0	0	0	1	0	0	0	1	0	0	0
Waycross	1	0	0	0	0	0	1	0	0	0	0	0	0	0
:z13,z28:I,z13,z28	1	0	0	0	0	0	0	0	0	0	0	0	0	0
Total	74	7	36	37	7	37	53	44	16	9	59	1	42	56.8

* AMP, ampicillin; GEN, gentamicin; KAN, kanamicin; CTX, cefotaxim; CIP, ciprofloxacin; SUL, sulphonamides; TET, tetracycline; CHL, chloramphenicol; TMP, trimethoprim; NAL, nalidixic acid; MEM, meropenem. ** MDR: multidrug-resistant strain. Susceptibility to fosfomycin and macrolides was not phenotypically analyzed, but one *S.* Menston strain contained a fosfomycin-resistant gene (*fosA7*), while *S.* Kentucky, *S.* Larochelle and *S.* Chester were all predicted to have macrolide resistance genes (*erm(A)*, *erm(B)* and *mph(A)*]. Notably, the meropenem-resistant *S.* Larochelle strain carried *qnrB19* and *erm (B)* genes, unrelated to the meropenem resistance, while point mutations were observed in the *ompR* gene of this strain, possibly explaining the resistance to meropenem.

**Table 2:** Distribution of resistance genes in *Salmonella* serovars based on in silico prediction.

		AMP	GEN	KAN	CIP	NAL	SUL	TMP	TET	CHL
Serovar	No	*bla_TEM_*	*aac(3)-Id aac(3)-IIa aac(3)-Iva*	*aac(6′)-Iaa, aph(3′)-Ia,b*	*T57S:S80I* and *S83F:D87Y*	*T57S:S80, S83F:D87Y*	*sul 1,2,3*	*dfrA (14, 15, 17)*	*tet(A) tet(M)*	*cat, cml, floR*
Abadina	2	0	0	2	0	2	0	0	0	0
Aberdeen	1	0	0	1	0	1	0	0	0	0
Alachua	1	0	0	1	0	1	0	0	0	0
Birmingham	1	0	0	1	0	0	0	0	0	0
Bradford	1	0	0	1	0	1	0	0	0	0
Chester *	2	0	0	2	0	1	0	0	0	0
Chomedey	1	0	0	1	0	0	1	0	1	0
Colindale	1	0	0	1	0	0	0	0	0	0
Corvalis	2	0	0	2	0	2	0	0	0	0
Essen	1	0	0	0	0	1	0	0	0	0
Give	1	0	0	1	1	1	0	0	0	0
Isangi	8	0	5	8	0	0	5	5	5	5
Ituri	2	0	0	2	0	2	0	0	0	0
Kentucky *	24	1	20	23	21	21	21	1	21	1
Larochelle *	4	0	0	0	0	4	0	0	0	0
Menston ^§^	1	0	0	1	0	1	0	0	0	0
Muenster	4	0	0	4	3	3	3	0	3	0
Poona	1	0	0	1	0	0	0	0	0	0
Schwarzengrund	4	3	3	3	0	3	3	0	4	0
Takoradi	6	0	0	6	1	5	0	0	0	0
Telelkebir	3	0	0	3	0	2	0	0	0	0
Virchow	1	0	0	1	0	0	0	0	0	0
Waycross	1	0	0	1	0	0	0	0	0	0
:z13,z28:I,z13,z28	1	0	0	1	0	1	0	0	0	0
Total	74	4	28	70	26	52	33	6	34	6

^§^*fosA7* (fosfomycin-resistant gene), * *erm (A), erm (B), mph (A), mef* macrolide resistance. AMP, ampicillin; GEN, gentamicin; KAN, kanamicin; CIP, ciprofloxacin; SUL, sulphonamides; TET, tetracycline; CHL, chloramphenicol; TMP, trimethoprim; NAL, nalidixic acid.

**Table 3:** Comparison between in silico plasmid prediction and detection of plasmids by gel electrophoresis.

Serovar and Strains	Plasmid Replicons	In Silico	* Base Pairs (kb)	# Electro-Phoresis	Plasmid Size (kb)
*S.* Isangi					
A18	Col(pHAD28)	+	2.7	+	2.6
A34	Col(pHAD28), Col440I	+/+	2.7/2.5	+/+	2.6/2
A54	Col(pHAD28)	+	2.7	+	2.6
C15	Col(pHAD28)	+	2.7	+	2.6
C24	Col(pHAD28)	+	2.7	+	2.6
C46	Col(pHAD28), IncHI2/IncHI2A	+/+	2.7/14.5	+/-	2.6/ND
*S.* Kentucky					
A16	IncFIIpCRY	+	41	+	36
A39	ColpVC	+	1.9	+	2
A40	IncFIIpCRY	+	41	+	36
A56	IncFIIpCRY	+	41	+	36
A61	ColpVC	+	1.9	+	2
A69	ColpVC	+	1.9	+	2
B14	ColpVC	+	1.9	+	2
B17	IncM1	+	61	+	63
C29	Col(pHAD28)	+	2.7	-	ND
C36	IncHI2A	+	14.5	-	ND
C44	IncFIIpCRY	+	41	+	36
*S.* Larochelle					
C33	Col(pHAD28)	+	2.7	+	2.6
C34	Col(pHAD28), Col(Ye4449)	+/+	2.7/4.2	+/+	2.6/3.5
C37	Col(pHAD28), Col(Ye4449)	+/+	2.7/4.2	+/+	2.6/3.5
C50	Col(pHAD28), Col(Ye4449)	+/+	2.7/4.2	+/+	2.6/3.5
*S.* Menston					
C10	IncI1 gamma	+	88	-	ND
*S.* Muenster					
A28	IncQ1	+	12	+	14
A55	IncQ1	+	12	-	ND
A59	IncQ1	+	12	+	14
*S.* Schwarzengrund					
A5	IncN	+	56	+	62
A71	IncN	+	56	+	62
*S.* Takoradi					
B21	IncL	+	55	+	62
B28	IncL	+	55	+	62
B36	IncL	+	55	+	62

ND = not detected, +/- indicates presence or absence, * plasmid size in base pair as predicted by in silico, # plasmid size in base pairs as detected by electrophoresis.

**Table 4:** Pathogenicity island content of *Salmonella* isolates from commercial poultry farms in Nigeria.

Serovars	Number	ST Type	SPI Profile
*S.* Abadina	2	3899	SPI-1 *, C63PI
*S.* Aberdeen	1	3320	SPI-1, C63PI C63PI SPI-3, SPI-5, SPI-13, SPI-14, C63PI
*S.* Alachua	1	7743	SPI-1, SPI-2, SPI-3, SPI-4, C63PI
*S.* Birmingham	1	7749	SPI-3, C63PI
*S.* Bradford	1	7746	SPI-1, SPI-2, SPI-3, C63PI
*S.* Chester	2	441	SPI-1, SPI-2, SPI-3, SPI-4 *, SPI-13, SPI-14, C63PI
*S.* Chomedey	1	3961	SPI-1, SPI-3, SPI-4, C63PI
*S.* Colindale	1	584	SPI-1, SPI-3, SPI-13, SPI-14, C63PI
*S.* Corvalis	2	7744	SPI-1 *, SPI-2 *, SPI-3, SPI-4 *, SPI-5, C63PI
*S.* Essen	1	7747	SPI-1, SPI-3, C63PI
*S.* Give	1	524	SPI-1, SPI-2, SPI-3, SPI-4, SPI-13, SPI-14, C63PI
*S.* Isangi	8	216	SPI-1 ^§^, SPI-2 ^§^, SPI-3 ^§^, SPI-4, SPI-5, SPI-13, SPI-14, C63PI ^§^
*S.* Ituri	2	4498	SPI-1, SPI-2 ^§^, SPI-3, SPI-5 ^§^, C63PI ^§^
*S.* Kentucky	24	198	SPI-1, SPI-2, SPI-3, SPI-4, SPI-14, C63PI ^§^
*S.* Larochelle	4	22	SPI-1, SPI-3, SPI-4, SPI-13 ^§^, SPI-14 ^§^, C63PI ^§^
*S.* Menston	1	7742	SPI-1, SPI-2, C63PI
*S.* Muenster	4	321	SPI-1 ^§^, SPI-2 *, SPI-13, SPI-14, C63PI
*S.* Poona	1	308	SPI-1, SPI-13, SPI-14, C63PI
*S.* Schwarzengrund	4	96	SPI-2, SPI-3 ^§^, SPI-4, SPI-13, SPI-14, C63PI
*S.* Takoradi	6	531	SPI-1, SPI-2, SPI-3, SPI-4, SPI-5, SPI-13 ^§^, C63PI ^§^
*S.* Telelkebir	3	2222	SPI-1 *, SPI-2 *, SPI-3 *, C63PI ^§^
*S.* Virchow	1	6166	SPI-1, SPI-2, SPI-3, SPI-4, SPI-13, SPI-14
*S.* Waycross	1	7745	SPI-1, SPI-2, SPI-3, SPI-4, SPI-8, C63PI
:z13,z28:I,z13,z28	1	-	SPI-3, SPI-5, C63PI

* present in some strains; ^§^ present in most strains.

## Data Availability

Publicly available datasets were analyzed in this study. This data can be found here: [https://www.ebi.ac.uk/ena/ PRJEB37477].

## Supplementary Materials

The following are available online at https://www.mdpi.com/2079-6382/10/2/99/s1. Files S1–S4: Table S1. Relationship between phenotypic and genotypic antimicrobial resistance profiles of *Salmonella* serovars obtained from commercial poultry farms in North West Nigeria. Table S2A. Concordance between phenotypic (CLSI standards) and genotypic antimicrobial resistance predictions, Table S2B. Concordance between phenotypic (EUCAST ECOFFs) and genotypic antimicrobial resistance predictions, Figure S1. Stop codon insertion in the amino acid sequence in the regulon upstream of the *aac (6)-Iaa* gene of isolates. Figure S2. Distribution of zone diameter from the breakpoints and prediction of resistant genes. Figure S3. Plasmid profiles of some selected *Salmonella* serovars. Figure S4A. Alignment using MAUVE of the ColpVC plasmid of *Salmonella* Kentucky to the reference plasmid CP047459.1. Figure S4B. Alignment using MAUVE of the IncFIIpCRY plasmid of *Salmonella* Kentucky to the reference plasmid CP022119.1. Figure S4C. Alignment using MAUVE of the IncL plasmid of *Salmonella* Takoradi to the reference plasmid CP018461.1. Figure S5. Workflow of plasmid reconstruction using a combination of approaches.
