# Supplementary material for: Genomic Analysis of Antimicrobial Resistance and Resistance Plasmids in Salmonella Serovars from Poultry in Nigeria
Source: Antibiotics (Basel). 2021 Jan 20;10(2):99. doi: 10.3390/antibiotics10020099 (PMC7909428; doi:10.3390/antibiotics10020099)
Supplement: Supplementary file 1 [file antibiotics-10-00099-s001.zip › Supplementary/Supplementary files.docx]

**Table S1.** Relationship between phenotypic and genotypic antimicrobial resistance profile of *Salmonella* serovars obtained from commercial poultry farms in North West, Nigeria.

| **Strain ID** | **Serovar** | **Phenotypic profile** | **Resistant genes**  **and point mutations** | **Plasmid replicons** | **Resistant genes on plasmid** |
| --- | --- | --- | --- | --- | --- |
| A5 | Schwarzengrund | AMP-GEN-CTX-CIP-SSS-TCY- NAL | *aac (3)-IIa*, *aac (6’)-Iaa*, *aph (3’’)-Ib*, *aph (6’)-Id*, *bla*_TEM-215_, *qnrS1*, *tet(A)*, *sul2*, (T57S:S80I) | IncN | *bla*_TEM_, *aac(3)-II-X*, *aph(3’’)-I*, *aph(6)-Ic/aph(6)*, *tet(R)*, *tet(A)*, *sul2*, *qnr*, DMT superfamily |
| A12 | Kentucky | CTX-KAN-CIP | *aac (3)-Id*, *aac (6’)-Iaa*, *aadA7*, *aph (3’’)-Ib*, *aph (6’)-Id*, *tet(A)*, *sul 1* |  |  |
| A15 | Kentucky | GEN-KAN-CIP-SSS-TCY- NAL | *aac (3)-Id*, *aac (6’)-Iaa*, *aadA7*, *aph (3’’)-Ib*, *aph (6’)-Id*, *tet(A)*, *sul 1*, (T57S:S80I), (S83F:D87Y) |  |  |
| A16 | Kentucky | CTX-KAN-CIP-SSS-TCY- NAL | *aac (3)-Id*, *aac (6’)-Iaa*, *aadA7*, *aph (3')-Ia*, *aph (3’’)-Ib*, *aph (6’)-Id*, *tet(A)*, *sul1*, (T57S:S80I), (S83F:D87Y ) | IncFIIpCRY | No resistant gene located |
| A18 | Isangi | GEN-CTX-KAN-CIP-TCY-CHL-TMP-NAL | *aac (3)-VIa*, *aac (6’)-Iaa*, *aadA1*, *aadA5*, *aph (3’)-Ia*, *dfrA15*, *dfrA17*, *floR*, *qnrB19*, *tet(A)*, *sul1* | Col(pHAD28), IncHI2, IncHI2A | *sul2*, *aph(3’’)-I*, *aph(6)-Ic/aph(6)*-Id, *tet(R)*, *Tet(A)*, *qnrB19*, DMT superfamily |
| A24 | Colindale | CTX-CIP-SSS-CHL | *aac (6’)-Iaa* |  |  |
| A25 | Chester | CTX-KAN-CIP-SSS | *aac (6’)-Iaa* |  |  |
| A27 | Aberdeen | CTX-CIP-SSS-TCY-NAL | *aac (6’)-Iaa*, (T57S:S80I) |  |  |
| A28 | Muenster | CTX-CIP-SSS-TCY-CHL-NAL | *aac (6’)-Iaa, aph (3’’)-Ib, aph (6)- Id, sul2, tet(A),* (T57S:S80I)*,* (S83F:D87Y ) | IncQ1 | *aph (3")-I, aph(6)-Id,* *tet(R)*, *tet(A)*, DMT superfamily |
| A29 | Kentucky | GEN-CTX-KAN-SSS-TCY-NAL | *aac (6’)-Iaa*, *aac (3)-Id*, *aadA7*, *aph (3’)-Ia*, *aph (3´´)-Ib*, *aph (6’)-Id*, *tet(A)*, *sul 1,* (T57S:S80I)*,* (S83F:D87Y ) |  |  |
| A31 | Schwarzengrund | AMP-GEN-CIP-SSS-TCY-CHL-NAL | *aac (3)-IIa*, *aac (6’)-Iaa*, *aph (3’’)-Ib*, *aph (6’)-Id*, *bla*_TEM-215_, *qnrS1*, *tet(A)*, *sul2*, (T57S:S80I) |  |  |
| A32 | Give | NAL | *aac (6’)-Iaa*, (T57S:S80I), (S83F:D87Y ) |  |  |
| A34 | Isangi | CIP-NAL | *aac (6’)-Iaa*, *qnrB19* | Col(pHAD28), Col440I | *qnrB19* |
| A35 | Isangi | CIP-NAL | *aac (6’)-Iaa*, *qnrB19* |  |  |
| A37 | Chester | GEN-KAN-SSS-TCY- NAL | *aac (6’)-Iaa*, (T57S:S80I) |  |  |
| A39 | Kentucky | GEN-KAN-CIP-SSS-TCY-NAL | *aph (3’’)-Ib*, *aph (6)- Id*, *aadA7*, *aac (3)-Id*, *aac (6’)-Iaa*, *tet(A)*, *sul1*, (S83F:D87Y ), (T57S:S80I) | ColpVC | No resistant gene located |
| A40 | Kentucky | CTX-KAN-CIP-SSS-TCY-CHL-NAL | *aac (3)-Id*, *aac (6’)-Iaa*, aadA7, *aph (3’)-Ia*, *aph (3’’)-Ib*, *aph (6’)-Id*, *tet(A)*, *sul1*, (S83F:D87Y ), (T57S:S80I) | IncFII(pCRY) | No resistant gene located |
| A44 | Virchow | CTX-CIP-SSS-NAL | *aac (6’)-Iaa* |  |  |
| A48 | Ituri | CIP-SSS-NAL | *aac (6’)-Iaa*, *qnrB19*, (T57S:S80I) |  |  |
| A51 | Schwarzengrund | AMP-GEN-KAN-CIP-SSS-TCY-CHL-NAL | *aac (6’)-Iaa*, *qnrS1*, *tet(A)*, (T57S:S80I) |  |  |
| A52 | Waycross | CTX-CIP-SSS | *aac (6’)-Iaa* |  |  |
| A53 | Ituri | CIP-SSS-TCY-NAL | *aac (6’)-Iaa*, *qnrB19*, (T57S:S80I) |  |  |
| A54 | Isangi | CIP-SSS-TCY-CHL-TMP-NAL | *aac (3)-VIa*, *aac (6’)-Iaa*, *aadA1*, *aadA5*, *aph (3’)-Ia*, *dfrA15*, *dfrA17*, *floR*, *qnrB19*, *tet(A)*, *sul1* | Col(pHAD28) | *qnrB19* |
| A55 | Muenster | KAN-CIP-SSS-TCY-NAL | *aac (6’)-Iaa*, *aph (3’’)-Ib*, *sul2*, *tet(A)*, (T57S:S80I), (S83F:D87Y ) | IncQ1 | *aph(6)-Ic/aph(6)-Id*, *aph(3’’)-I*, *sul2*, *tet(R)*, *tet(A)*, DMT superfamily |
| A56 | Kentucky | GEN-KAN-CIP-SSS-TCY-NAL | *aac (3)-Id*, *aac (6’)-Iaa*, *aadA7*, *aph (3’)-Ia*, *aph (3’’)-Ib*, *aph (6)- Id*, *tet(A)*, *sul1*, (S83F:D87Y ), (T57S:S80I) | IncFIIpCRY | No resistant gene located |
| A59 | Muenster | CTX-CIP-SSS-TCY-NAL | *aac (6’)-Iaa*, *aph (3’’)-Ib*, *aph (6’)-Id*, *tet(A)*, *sul2* | IncQ1 | *aph(6)-Ic/aph(6)-Id*, *aph(3’’)-I*, s*ul2*, *tet(R)*, *tet(A)*, DMT superfamily |
| A61 | Kentucky | GEN-KAN-CIP-SSS-TCY-NAL | *aac (6’)-Iaa*, (S83F:D87Y ), (T57S:S80I) | ColpVC | No resistant gene located |
| A69 | Kentucky | GEN-KAN-CIP-SSS-TCY-NAL | *aac (3)-Id*, *aac (6’)-Iaa*, *aadA7*, *aph (3’’)-Ib*, *aph (6’)-Id*, *tet(A)*, *sul1*, (T57S:S80I), (S83F:D87Y ) | ColpVC | No resistant gene located |
| A71 | Schwarzengrund | AMP-GEN-KAN-CIP-SSS-TCY-NAL | *aac (3)-IIa*, *aac (6’)-Iaa*, *aph (3’’)-Ib*, *aph (6’)-Id*, *bla*_TEM-215_, *qnrS1*, *tet(A)*, *sul2*, (T57S:S80I) | IncN | *bla*_TEM_, *aac(3)-II-X*, *tet(R)*, *tet(A)*, DMT superfamily, s*ul2*, *aph(3’’)-I*, *aph(6)-Ic/aph(6)*-Id |
| A72 | Muenster | CTX-CIP-SSS-NAL | *aac (6’)-Iaa*, (T57S:S80I), (S83F:D87Y ) |  |  |
| B4 | Kentucky | GEN-CTX-KAN-CIP-SSS-TCY-NAL | *aac (3)-Id*, *aac (6’)-Iaa*, *aadA7*, *aph (3’)-Ia* *aph (3’’)-Ib*, *aph (6)- Id*, *qnrB19*, *sul1*, *tet(A)*, (T57S:S80I), (S83F:D87Y ) |  |  |
| B5 | Kentucky | GEN-KAN-SSS-TCY-NAL | *aac (3)-Id*, *aac (6’)-Iaa*, *aadA7*, *aph (3’’)-Ib*, *aph (6)- Id*, *sul1*, *tet(A)*, (T57S:S80I), (S83F:D87Y ) |  |  |
| B12 | Takoradi | CTX-KAN-SSS | *aac (6’)-Iaa*, (T57S:S80I), (S83F:D87Y ) |  |  |
| B13 | Poona | GEN-CIP-SSS | *aac (6’)-Iaa* |  |  |
| B14 | Kentucky | GEN-KAN-CIP-SSS-TCY-NAL | *aac (3)-Id*, *aac (6’)-Iaa*, *aadA7*, *aph (3’’)-Ib*, *aph (6)- Id*, *sul1*, *tet(A)* | ColpVC | No resistant gene located |
| B16 | Kentucky | GEN-CIP-SSS-TCY-NAL | *aac (3)-Id*, *aac (6’)-Iaa*, *aadA7*, *aph (3’’)-Ib*, *aph (6)- Id*, *sul1*, *tet(A)*, (S83F:D87Y ), (T57S:S80I) |  |  |
| B17 | Kentucky | GEN-CIP-SSS-TCY-NAL | *aac (3)-Id*, *aac (6’)-Iaa*, *aadA7*, *aph (3’’)-Ib*, *aph (6)- Id*, *sul1*, *tet(A)*, (S83F:D87Y ), (T57S:S80I) | IncM1 | QacE delta 1, *aac(3)-I, ant(3'')-Ia*, *sul2*, *aph(6)-Ic/aph(6)*-Id, *aph(3’’)-I*, *tet(R)*, *tet(A)*, |
| B20 | Takoradi | GEN-KAN-CIP-SSS-TCY-NAL | *aac (6’)-Iaa*, (T57S:S80I) |  |  |
| B21 | Takoradi | CIP | *aac (6’)-Iaa* | IncL | No resistant gene located |
| B26 | Kentucky | GEN-CTX-KAN-CIP-SSS-TCY-CHL -NAL | *sul1*, *tet(A)*, *qnrB19*, *aph (3’)-Ia* *aph (3’’)-Ib*, *aph (6)- Id*, *aadA7*, *aac (3)-Id*, *aac (6’)-Iaa*, (S83F:D87Y ), (T57S:S80I) |  |  |
| B27 | Corvallis | CTX-CIP | *aac (6’)-Iaa*, (T57S:S80I) |  |  |
| B28 | Takoradi | CIP-SSS | *aac (6’)-Iaa*, (T57S:S80I) | IncL | No resistant gene located |
| B31 | Takoradi | KAN-CIP | *aac (6’)-Iaa*, (T57S:S80I) |  |  |
| B35 | Kentucky | GEN-KAN-CIP-SSS-TCY-NAL | *aac (3)-Id*, *aac (6’)-Iaa*, *aadA7*, *aph (3’’)-Ib*, *aph (6’)-Id*, *tet(A)*, *sul1*, (S83F:D87Y ), (T57S:S80I) |  |  |
| B36 | Takoradi | CTX-KAN-CIP-SSS | *aac (6’)-Iaa*, (T57S:S80I) | IncL | No resistant gene located |
| C1 | Kentucky | GEN-CIP-SSS-TCY-NAL | *aac (3)-Id*, *aac (6’)-Iaa*, *aadA7*, *aph (3’’)-Ib*, *aph (6’)-Id*, *tet(A)*, *sul1*, (T57S:S80I), (S83F:D87Y ) |  |  |
| C3 | Chomedey | CIP-SSS-TCY | *aac (6’)-Iaa*, *aph (3’’)-Ib*, *aph (6’)-Id*, *qnrB19*, *tet(A)*, *sul2* |  |  |
| C5 | Alachua | CIP-NAL | *aac (6’)-Iaa*, *qnrB19*, (T57S:S80I) |  |  |
| C6 | Essen\|Hato | KAN-CIP-SSS-TCY-CHL-NAL | *aac (6’)-Iaa*, *qnrB19*, (T57S:S80I) |  |  |
| C10 | Menston | CIP-TCY-CHL-TMP-NAL | *aac (6’)-Iaa*, *fosA7*, (T57S:S80I) | IncI1 gamma | No resistant gene located |
| C11 | Isangi | GEN-CIP-NAL | *aac (6’)-Iaa*, *qnrB19* |  |  |
| C12 | Bradford | CTX-CIP-SSS-NAL | *aac (6’)-Iaa*, *qnrB19*, (T57S:S80I) |  |  |
| C13 | Kentucky | AMP-GEN-CTX-KAN-CIP-SSS-TCY -NAL | *aac (3)-Id*, *aac (6’)-Iaa*, *aadA7*, *aph (3’)-Ia*, *aph (6)- Id*, *sul1*, *tet(A)*, (S83F:D87Y ), (T57S:S80I) |  |  |
| C14 | Abadina | CTX-CIP-NAL | *aac (6’)-Iaa*, (T57S:S80I) |  |  |
| C15 | Isangi | GEN-CTX-CIP-SSS-TCY-CHL-TMP-NAL | *aac (3)-VIa*, *aac (6’)-Iaa*, *aadA1*, *aadA5*, *aph (3’)-Ia*, *dfrA15*, *dfrA17*, *floR*, *qnrB19*, *tet(A)*, *sul1* | Col(pHAD28) | *qnrB19* |
| C16 | Corvallis | GEN-KAN-CIP-SSS-TCY-NAL | *aac (6’)-Iaa*, *qnrB19*, (T57S:S80I) |  |  |
| C17 | Kentucky | AMP-GEN-CTX-KAN-CIP-SSS-TCY -NAL | *aac (3)-Id*, *aac (6’)-Iaa*, *aadA7*, *aph (3’)-Ia*, *aph (3’’)-Ib*, *aph (6)- Id*, *qnrB19*, *sul1*, *tet(A)*, (T57S:S80I), (S83F:D87Y ) |  |  |
| C24 | Isangi | GEN-CTX-CIP-SSS-TCY-CHL-TMP-NAL | *aac (3)-VIa*, *aac (6’)-Iaa*, *aadA1*, *aadA5*, *aph (3’)-Ia*, *dfrA15*, *dfrA17*, *floR*, *qnrB19*, *sul1*, *tet(A)* | Col(pHAD28) | *qnrB19* |
| C26 | Birmingham | CIP | *aac (6’)-Iaa* |  |  |
| C28 | :z13,z28:I,z13,z28 | CTX-CIP | *aac (6’)-Iaa*, (T57S:S80I) |  |  |
| C29 | Kentucky | GEN-KAN-CIP-SSS-TCY-NAL | *aac (6’)-Iaa*, *aac (3)-VIa*, *aph (3’)-Ia*, *aadA1*, *aadA5*, *qnrB19*, *sul1*, *tet(A)*, (T57S:S80I), (S83F:D87Y ) | Col(pHAD28) | *qnrB19* |
| C30 | Kentucky | GEN-KAN-CIP-SSS-TCY-NAL | *aac (6’)-Iaa*, *aac (3)-Id*, *aadA7*, *aph (3’)-Ia* *aph (3’’)-Ib*, *aph (6)- Id*, *qnrB19*, *sul1*, *tet(A)*, (T57S:S80I), (S83F:D87Y ) |  |  |
| C31 | Kentucky | GEN-CTX-KAN-CIP-NAL | *aac (6’)-Iaa*, (T57S:S80I), (S83F:D87Y ) |  |  |
| C33 | Larochelle | CTX-CIP-TCY-CHL-MEM-TMP-NAL | *qnrB19*, erm (B), (T57S:S80I) | Col(pHAD28) | *qnrB19* |
| C34 | Larochelle | CTX-CIP-NAL | *aac (6’)-Iaa*, *qnrB19*, (T57S:S80I) | Col(pHAD28), Col(Ye4449) | *qnrB19* |
| C36 | Kentucky | AMP-GEN-CTX-KAN-CIP-SSS-TCY CHL-TMP-NAL | *aac (6’)-Iaa*, *aac (3)-Id*, *aadA1*, aad2b, *aph (3’)-Ia*, *aph (3’’)-Ib*, *aph (6)- Id*, *bla*_TEM-1B_, *catA1, cmlA1, dfrA14*, *floR*, *mef* *(B), mph (A),* *qnrS1*, *sul1, sul3*, *tet (A), tet (M)*, (S83F:D87Y ), (T57S:S80I) | IncHI2, IncHI2A | *tet(M),* *sul2*, aph(3')-I, *bla*_TEM_, *tet (R)*, *tet(A)*, DMT superfamily, *aph(6)-Ic/aph(6)*-Id, *aph(3’’)-I*, *mph* (A) family |
| C37 | Larochelle | CIP-NAL | *aac (6’)-Iaa*, *qnrB19*, (T57S:S80I) | Col(pHAD28), Col(Ye4449) | *qnrB19* |
| C39 | Abadina | CIP-NAL | *aac (6’)-Iaa*, (T57S:S80I) |  |  |
| C44 | Kentucky | GEN-KAN-CIP-SSS-TCY-NAL | *aac (3)-Id*, *aac (6’)-Iaa*, *aadA7*, *aph (3’’)-Ib*, *aph (6)- Id*, *sul1*, *tet(A)*, (S83F:D87Y ), (T57S:S80I) | IncFIIpCRY | No resistant gene located |
| C45 | Telelkebir | GEN-KAN-CIP-SSS-TCY-CHL-TMP-NAL | *aac (6’)-Iaa*, *qnrB19*, (T57S:S80I) |  |  |
| C46 | Isangi | GEN-KAN-CIP-SSS-TCY-CHL-TMP-NAL | *aac (3)-VIa*, *aac (6’)-Iaa*, *aadA1*, *aadA5*, *aph (3’)-Ia*, *dfrA15*, *dfrA17*, *floR*, *qnrB19*, *tet(A)*, *sul1* | Col(pHAD28), IncHI2, IncHI2A | SMR protein, *qnrB19* |
| C50 | Larochelle | GEN-CTX-KAN-CIP-SSS-MEM-NAL | *aac (6’)-Iaa*, *qnrB19*, (T57S:S80I) | Col(pHAD28), Col(Ye4449) | *qnrB19* |
| C52 | Telelkebir | CTX-KAN-CIP-SSS-NAL | *aac (6’)-Iaa*, *qnrB19*, (T57S:S80I) |  |  |
| C53 | Telelkebir | CIP | *aac (6’)-Iaa*, *qnrB19* |  |  |

**Phenotypic resistance**: AMP, ampicillin; CHL, chloramphenicol; CIP, Ciprofloxacin; CTX, Cefotaxime; GEN, gentamicin; KAN, kanamycin; MEM, meropenem; NAL, Nalidixic acid; SUL, Sulphonamides; TET, tetracycline; TMP, trimethoprim

**Resistance genes**

Aminoglycoside resistance; *aac (3)-IIa, aac, (6')-Iaa, aph (3'')-Ib, aph (6')-Id, ant(6)-Ia, ant (9)-Ia, aph (3')-III*, Beta lactamase; *bla*_TEM_*_-215_*, *bla*_TEM-1B_ Fluoroquinolones resistance; (*qnrS, qnrB19*), Tetracycline resistance; *tet(A), tet (M), (R)*; Florefenicol resistance; *floR*, Fosfomycin: *fosA7*, Chloramphenicol resistance; *catA1, cmlA1*, Trimethoprim resistance; *dfrA14*, *dfrA15, dfrA17*, Sulphonamides resistance; s*ul1, sul2,sul3*, Macrolide resistance; *erm (B), erm (A), mef (B), mph (A)*, Pentapeptide repeat protein *qnrB* family, Permease of the drug metabolite trasnsporter (DMT) superfamily, Small multidrug resistance (SMR) efflux transporter; QacE delta 1, quaternary ammonium compounds, Multidrug efflux pump MdtL (of MFS type).

**Table S2A**. Concordance between phenotypic (CLSI standards) and genotypic antimicrobial resistance predictions.

| **Antimicrobial class** | **Phenotype** | | **Genotype** | | | |  |
| --- | --- | --- | --- | --- | --- | --- | --- |
|  | n | (%) | Resistance genes and point mutations | n | (%) | Kappa | *p* value |
| **β- lactams** |  |  |  |  |  |  |  |
| Ampicillin | 7 | 9.5 | *bla_TEM_* | 4 | 5.4 | 0.70 | 0.0001 |
| Cefotaxime | 7 | 10.9 | Not predicted | 0 | 0.0 |  |  |
| **Aminoglycosides** |  |  |  |  |  |  |  |
| Gentamicin | 36 | 48.6 | *aac(3)-Id aac(3)-IIa aac(3)-IVa* | 28 | 37.8 | 0.62 | 0.0001 |
| Kanamycin | 37 | 50.0 | *aac (6')-Iaa, aph(3')-Ia,b* | 70 | 94.6 | 0.00 | 1.00 |
| **Fluoroquinolones** |  |  |  |  |  |  |  |
| Ciprofloxacin | 37 | 50.0 | S83F:D87Y and T57S:S80I | 26 | 35.1 | 0.52 | 0.0001 |
| Nalidixic acid | 59 | 79.7 | S83F:D87Y, T57S:S80I | 52 | 70.3 | 0.37 | 0.001 |
| **Folate Pathway Inhibitor** |  |  |  |  |  |  |  |
| Sulphonamides | 53 | 71.6 | *sul* (*1,2,3)* | 33 | 44.6 | 0.33 | 0.002 |
| Trimethoprim | 9 | 12.2 | *dfrA (14, 15, 17)* | 6 | 8.1 | 0.78 | 0.0001 |
| **Phenicols** |  |  |  |  |  |  |  |
| Chloramphenicol | 16 | 21.6 | *cat,cml,floR* | 6 | 8.1 | 0.49 | 0.0001 |
| **Tetracycline** |  |  |  |  |  |  |  |
| Tetracycline | 44 | 59.5 | *tet(A),tet (M)* | 34 | 45.9 | 0.68 | 0.0001 |

n= number

**Table S2**B. Concordance between phenotypic (EUCAST ECOFFs) and genotypic antimicrobial resistance predictions.

| **Antimicrobial class** | **Phenotype** | | **Genotype** | | | |  |
| --- | --- | --- | --- | --- | --- | --- | --- |
|  | n | (%) | Resistance genes and point mutations | n | (%) | Kappa | *p* value |
| **β- lactams** |  |  |  |  |  |  |  |
| Ampicillin | 9 | 12.5 | *bla_TEM_* | 4 | 5.4 | 0.58 | 0.0001 |
| Cefotaxime | 7 | 10.9 | Not predicted | 0 | 0.0 |  |  |
| **Aminoglycosides** |  |  |  |  |  |  |  |
| Gentamicin | 36 | 48.6 | *aac(3)-Id aac(3)-IIa aac(3)-IVa* | 28 | 37.8 | 0.62 | 0.0001 |
| Kanamycin | 37 | 50.0 | *aac (6')-Iaa, aph(3')-Ia,b* | 70 | 94.6 | 0.00 | 1.00 |
| **Fluoroquinolones** |  |  |  |  |  |  |  |
| Ciprofloxacin | 37 | 50.0 | S83F:D87Y and T57S:S80I | 26 | 35.1 | 0.52 | 0.0001 |
| Nalidixic acid | 59 | 79.7 | S83F:D87Y, T57S:S80I | 52 | 70.3 | 0.37 | 0.001 |
| **Folate Pathway Inhibitor** |  |  |  |  |  |  |  |
| Sulphonamides | 53 | 71.6 | *sul* (*1,2,3)* | 33 | 44.6 | 0.33 | 0.002 |
| Trimethoprim | 23 | 31.1 | *dfrA (14, 15, 17)* | 6 | 8.1 | 0.33 | 0.0001 |
| **Phenicols** |  |  |  |  |  |  |  |
| Chloramphenicol | 17 | 22.9 | *cat,cml,floR* | 6 | 8.1 | 0.46 | 0.0001 |
| **Tetracycline** |  |  |  |  |  |  |  |
| Tetracycline | 48 | 64.9 | *tet(A),tet (M)* | 34 | 45.9 | 0.58 | 0.0001 |


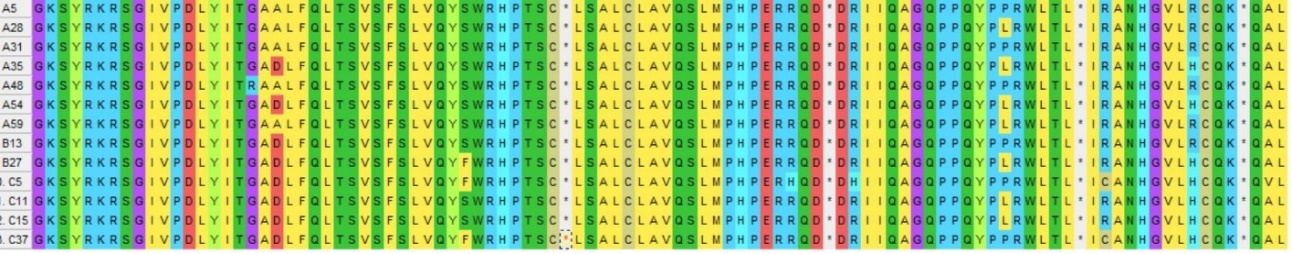

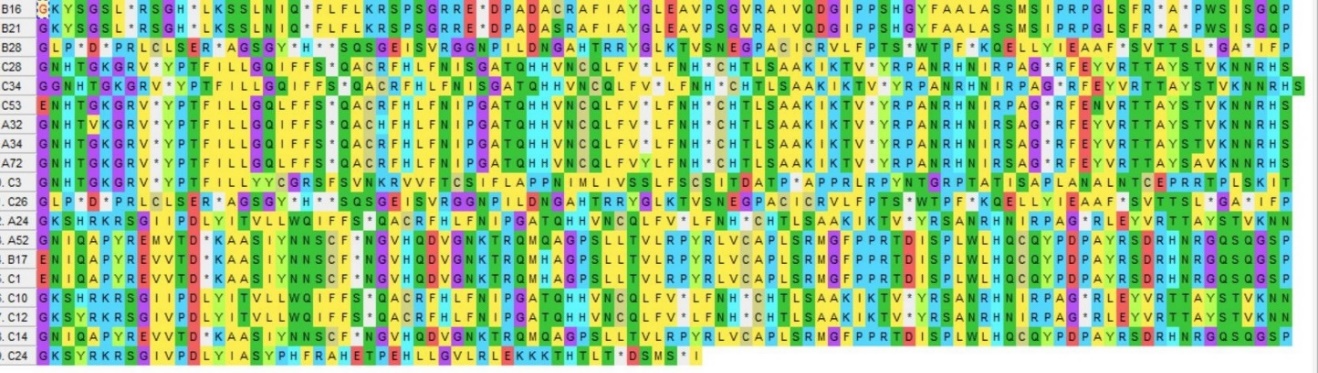


**Figure S1**. Stop codon insertion in the amino acid sequence in the regulon upstream of *aac (6) - Iaa* gene of isolates. * indicate stop codon using MEGA X v.10.2.2


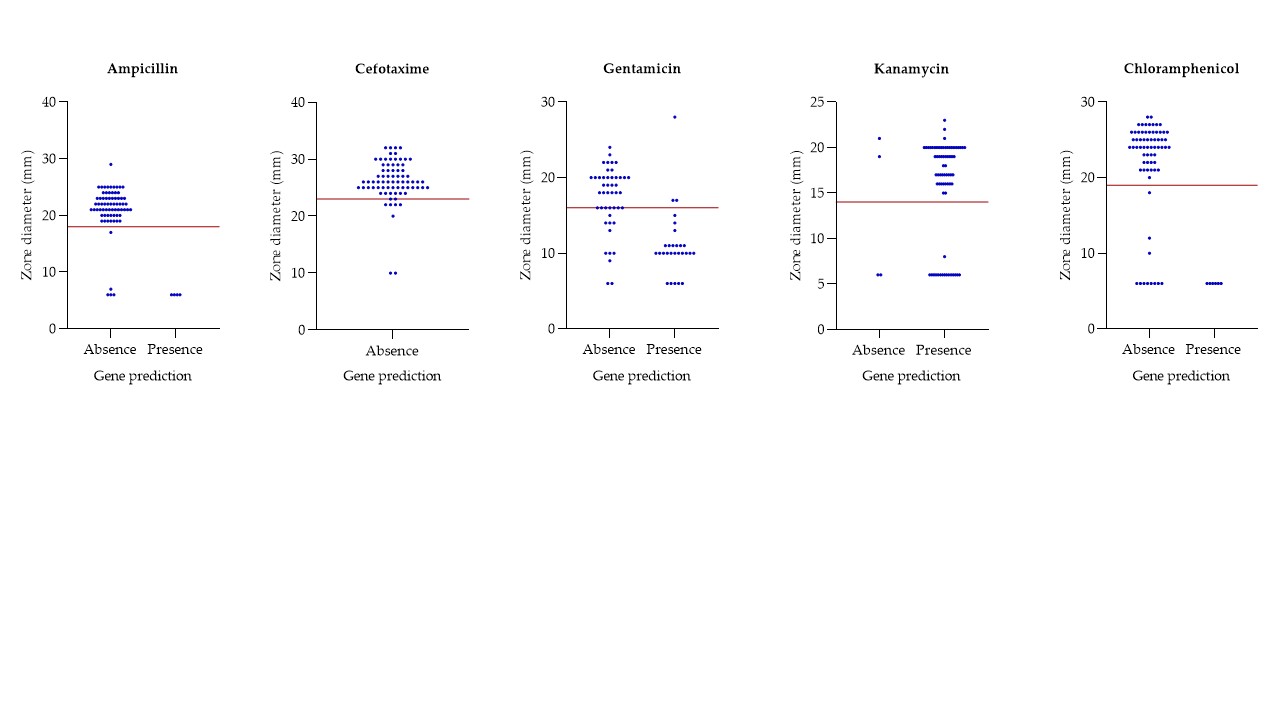

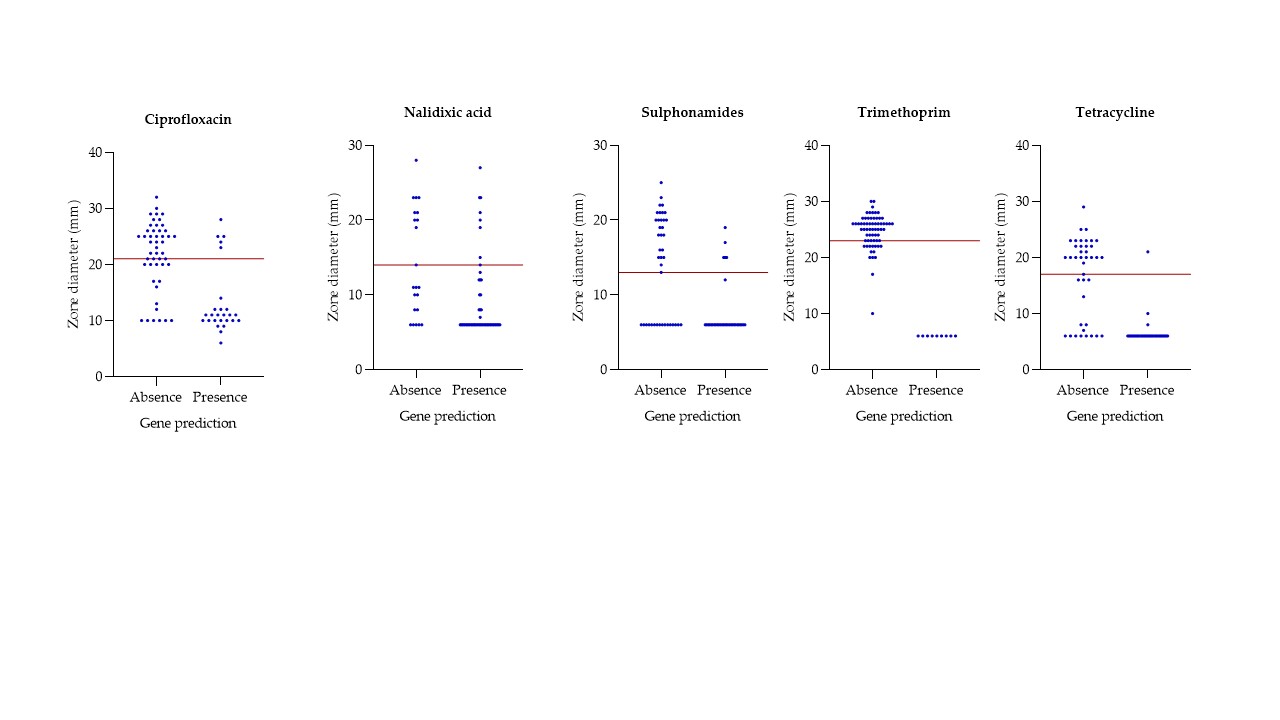


**Figure S2**. Distribution of zone diameter from the breakpoints and prediction of resistant genes using GraphPad v.8.0.1. The x-axis represent absence and presence resistance gene conferring resistance to that antibiotic. The y-axis represent zone diameters of strains. Each dark blue circle represents an individual observation of zone diameter from the clinical breakpoint. Observations below the breakpoints are phenotypically resistance, while those above are phenotypically sensitive. The red coloured horizontal y-axis intercept, represents the clinical breakpoints for that antibiotic.

**
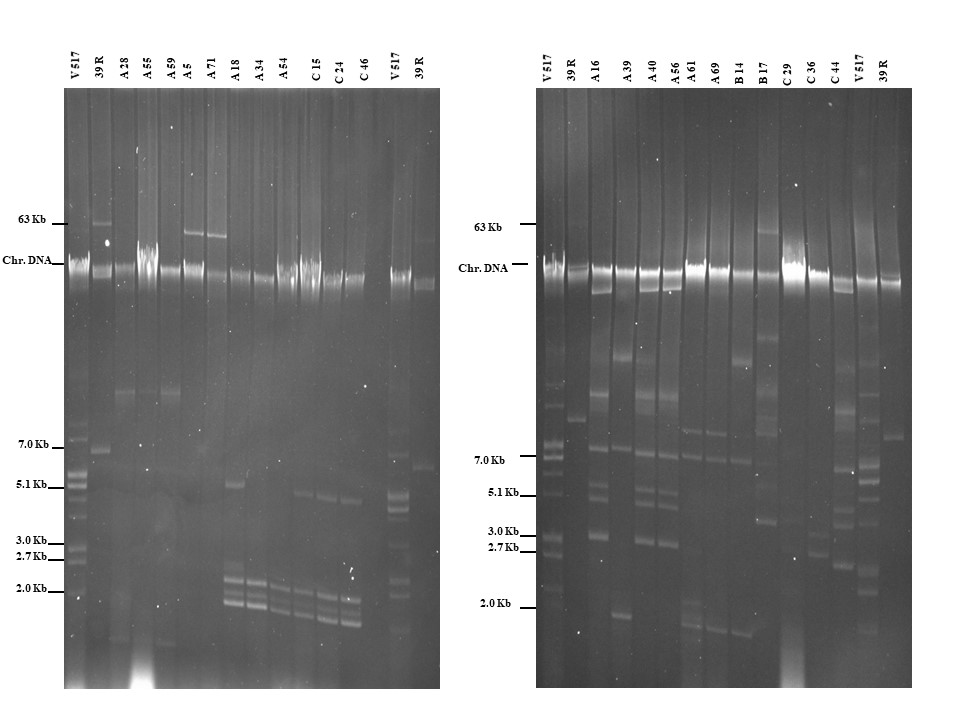
**

**Figure S3.** Plasmid profiles of some selected *Salmonella* serovars. Plasmid migration is based on the sizes of plasmids. Chr. is the chromosomal DNA of the strains. Lanes A28, A55, and A59 are IncQ1 plasmids from S. Muenster. Lanes A5 and A71 are IncN plasmids from *S.* Schwarzengrund. Lanes A18, A34, A54, C15, C24 and C46 are ColpHAD28 plasmids from *S.* Isangi. Lanes A16, A40, A56 and C44 are IncFIIpCRY of *S.* Kentucky, Lanes A39, A61, A69, and B17 are ColpVC plasmids of *S.* Kentucky. V 517 and 39 R are known markers of *E. coli* V517 and *E. coli* 39R861 strains. B17 IncM1 plasmid of *S.* Kentucky.


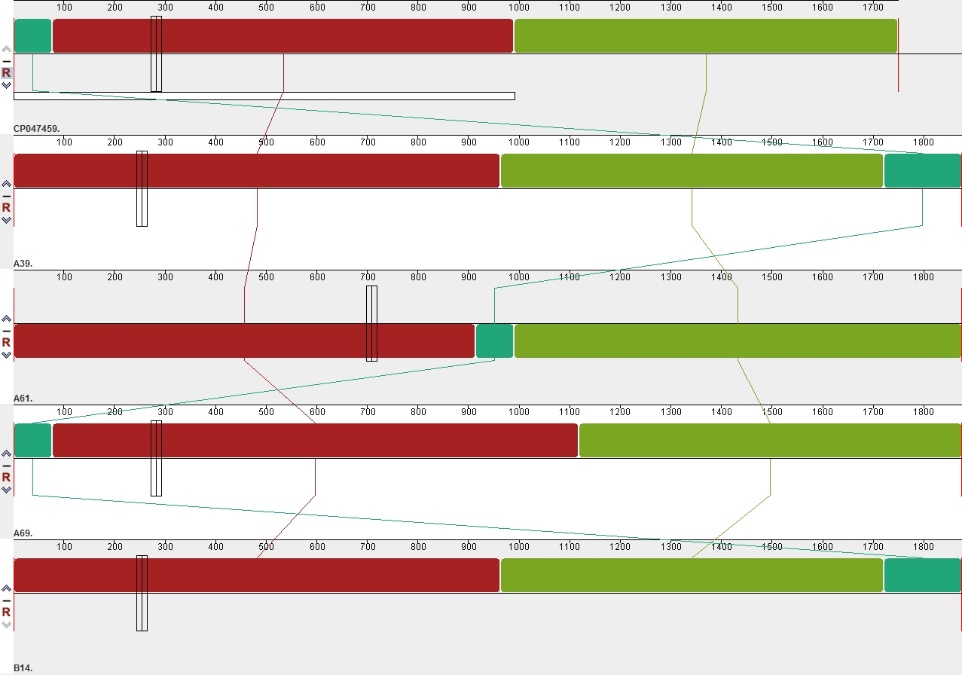


**Figure S4A.** Alignment using MAUVE of ColpVC plasmid of *Salmonella* Kentucky to the reference plasmid [CP047459.1](https://www.ncbi.nlm.nih.gov/nucleotide/CP047459.1?report=genbank&log$=nuclalign&blast_rank=1&RID=DAE48NMC014). The top row represent the reference plasmids, while the bottom four are plasmids from *S.* Kentucky from this study. Each of the coloured blocks represent a locally collinear block that is homologous that aligned to other part of other genomes

**
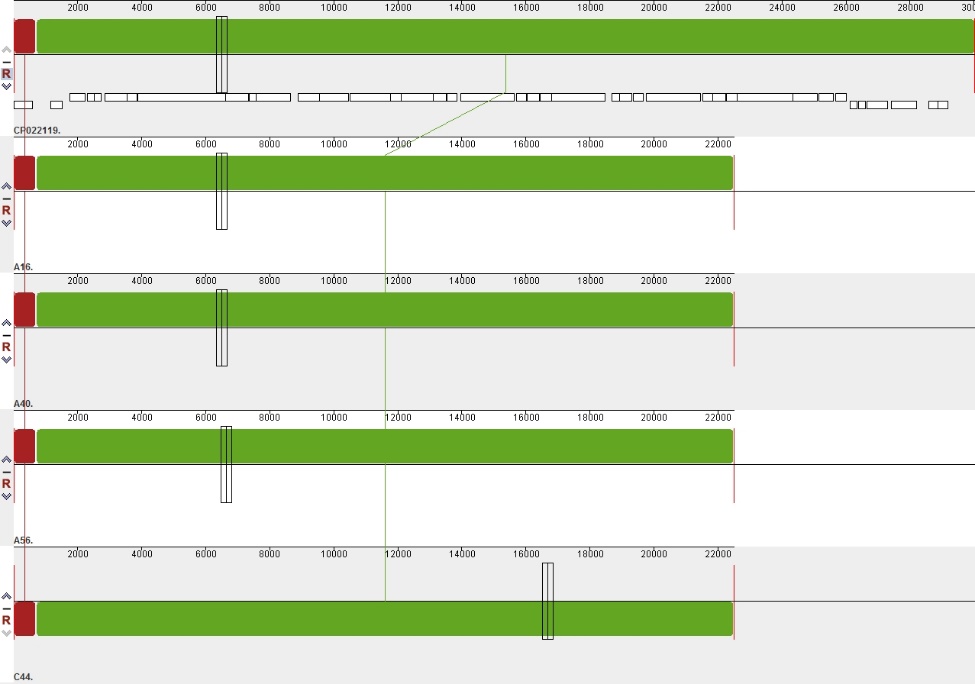
**

**Figure S4B.** Alignment using MAUVE of IncFIIpCRY plasmid of *Salmonella* Kentucky to the reference plasmid [CP022119.1](https://www.ncbi.nlm.nih.gov/nucleotide/CP022119.1?report=genbank&log$=nuclalign&blast_rank=14&RID=DAH58XAT01R) The top row represent the reference plasmids, while the bottom four are plasmids from *S.* Kentucky from this study. Each of the coloured blocks represent a locally collinear block that is homologous that aligned to other part of other genomes

**
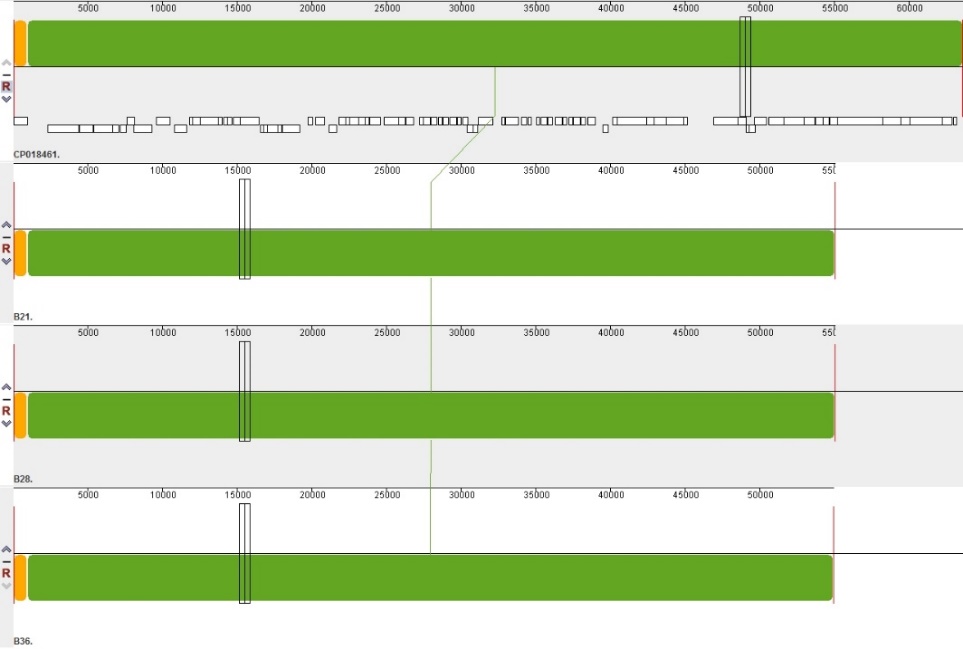
**

**Figure S4C.** Alignment using MAUVE of IncL plasmid of *Salmonella* Takoradi to the reference plasmid [CP018461.1](https://www.ncbi.nlm.nih.gov/nucleotide/CP018461.1?report=genbank&log$=nuclalign&blast_rank=9&RID=DCPZFYU4014) The top row represent the reference plasmids, while the bottom three are plasmids from *S.* Takoradi from this study. Each of the coloured blocks represent a locally collinear block that is homologous that aligned to other part of other genomes


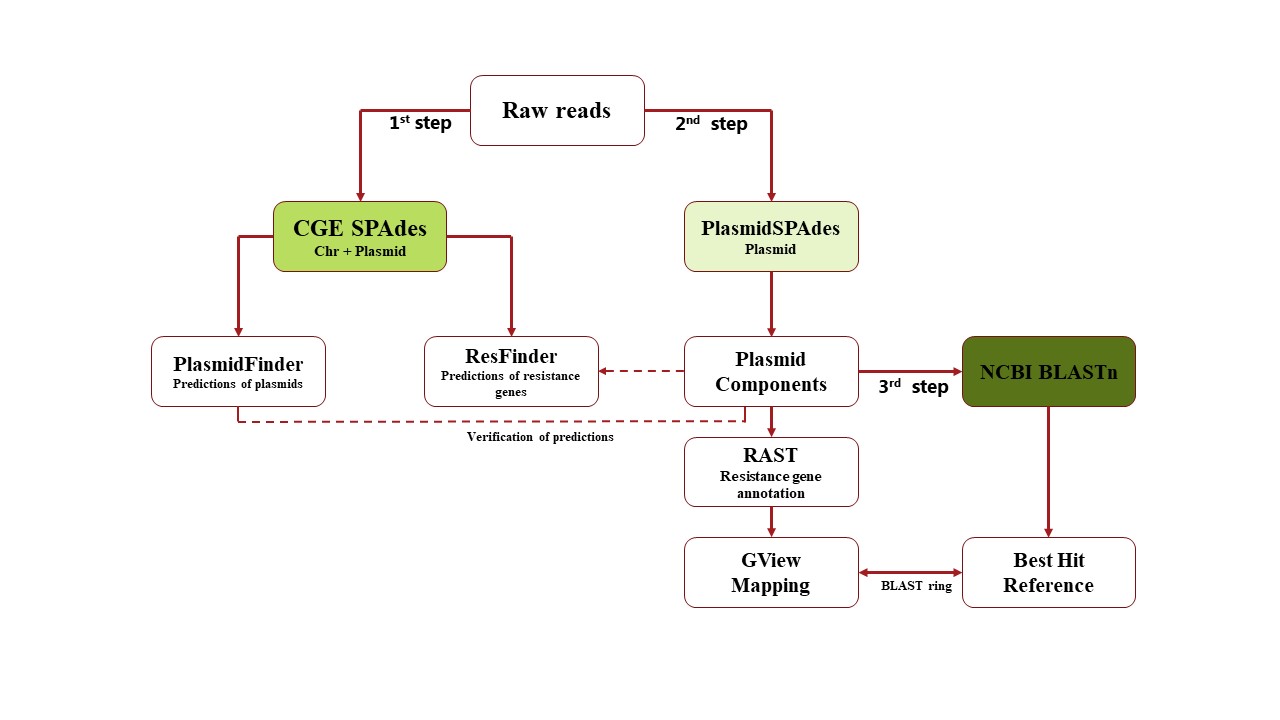


**Figure S5.** Workflow of plasmid reconstruction using combination of approaches. Different pipeline is presented in rectangular boxes. Light green colored rectangle indicate basic steps in the reconstruction. Dashed arrows represent second resubmission to the pipeline for verification.
